# Incidence, characteristics and suggestions for prevention of adverse events in supervised pediatric oncology exercise sessions

**DOI:** 10.3389/fped.2026.1809915

**Published:** 2026-04-29

**Authors:** Gabriele Gauß, Corina Silvia Rueegg, Christina Schindera, Stefan Meisegeier, Hannah Stalf, Thorben Sundermeier, Melisa Dietrich, Michael Maiwald, Annika Reus, Aleksandar Tomaskovic, Miriam Götte

**Affiliations:** 1 Department of Pediatrics III, Hematology and Oncology, University Hospital Essen, Essen, Germany; 2 Oslo Centre for Biostatistics and Epidemiology, Oslo University Hospital, Oslo, Norway; 3 Epidemiology, Biostatistics & Prevention Institute (EBPI), University of Zurich, Zurich, Switzerland; 4 Institute of Social and Preventive Medicine, University of Bern, Bern, Switzerland; 5 Division of Paediatric Oncology/Haematology, University Children's Hospital Basel, University of Basel, Basel, Switzerland; 6 Robert Koch Institute, Berlin, Germany; 7 DuMusstKämpfen gGmbH, Frankfurt, Germany; 8 Department of Rehabilitation and Sports Medicine, Institute of Sports Medicine, Hannover Medical School, Hannover, Germany; 9 DIPLOMA Private Hochschulgesellschaft mbH, Bad Sooden-Allendorf, Germany; 10 Department of Pediatrics, Goethe University Frankfurt, Frankfurt am Main, Germany; 11 West German Cancer Centre Essen, University Hospital Essen, Essen, Germany

**Keywords:** childhood cancer, CTCAE, health promotion, patient safety, physical activity, prevention, risk management

## Abstract

**Introduction:**

Supervised exercise sessions in pediatric oncology offer numerous benefits, including mitigated treatment-related symptoms and improved physical and psychological outcomes. However, systematic assessment of adverse events (AEs) remains limited which restricts implementation and optimization of exercise safety in this vulnerable population. We aimed to prospectively assess AEs during exercise sessions, describe their characteristics and provide recommendations for action to reduce AEs.

**Methods:**

A prospective observational study was conducted at six pediatric oncology centers over three years to systematically record AEs and related information occurring during usual care exercise sessions from acute cancer treatment to post-treatment phase. An AE was defined as any adverse event temporally associated with an exercise session, regardless of whether it was causally related to the exercise itself. Each AE was categorized and graded according to the Common Terminology Criteria for AEs, and additional contextual information was documented in a centralized database. Based on the findings, AE characteristics were analyzed and a multidisciplinary consensus process was used to develop recommendations.

**Results:**

In total, 178 (75% grade 1; 23% grade 2; 2% grade 3) AEs were documented across 74,083 supervised exercise sessions, corresponding to 1 AE per 416 sessions. No life-threatening AEs were observed. Of the 178 AEs, 85% (151/178) were judged as exercise-related, resulting in an incidence of 204 per 100,000 exercise sessions (0.2%). Most common AE types were pain (53%; *n* = 94), nausea or vomiting (20%; *n* = 35), and circulatory problems (17%; *n* = 30). Overall, AEs were primarily triggered by physical (over)exertion (63%; *n* = 112), medical treatments (44%; *n* = 78), and fall-related incidents (23%; *n* = 41). Based on these findings and existing guidelines, 11 recommendations for action to reduce AEs were developed, including a consensus-based risk assessment, multiprofessional collaboration, and continuous professional education of exercise experts providing the exercise sessions.

**Conclusion:**

We found an overall low incidence of AEs during supervised exercise sessions in pediatric oncology, of generally low grade. The study highlights the need for prospective studies to refine evidence-based prevention strategies, test their effectiveness and implement them.

## 1 Introduction

Supportive therapies in oncology have the potential to improve patients' physical and emotional well-being. Consequently, a variety of interventions are being implemented and evaluated in both adult and pediatric oncology settings (1, 2). Among these, supervised regular exercise sessions have gained particular prominence (3). Pediatric cancer encompasses a heterogeneous group of diseases, most commonly including leukemia, central nervous system tumors, lymphomas, and solid tumors such as neuroblastoma and sarcomas (4). This diagnostic heterogeneity is associated with diverse treatment regimens. Growing evidence supports the benefits of supervised regular exercise sessions in children and adolescents during active cancer treatment (5) and in the post-treatment phase (6), including improvements in muscle strength (7), endurance (8–10), functional mobility (11), gait function (12), immune function (13), cardio protection (14), and quality of life (15–17). Preliminary evidence also suggests reductions in cancer-related fatigue (8, 18, 19) and hospitalization time (20). Physical activity interventions during acute treatment seem to be of particularly important, as a decline in exercise capacity can be prevented through targeted exercise (5), thereby helping to reduce therapy- and inactivity-related side effects and support patients in coping with the physical burden of cancer treatment (21).

Against this background, the safety of supervised regular exercise sessions has become an increasing focus of attention. Current evidence in pediatric oncology suggests that targeted, supervised exercise during the acute treatment phase (1, 10, 22, 23) and in the post-treatment phase (24) is generally safe. However, no detailed registry or systematic, prospective assessment of adverse events (AEs) during supervised exercise sessions in pediatric oncology currently exists (25).

Only a small number of available supervised exercise trials document the occurrence of AEs in a systematic way, with many studies not providing details on how and when AEs were assessed or reported (26). Reviews usually indicate very few small of AEs in the included studies; however, it remains unclear whether AEs were absent or simply not recorded (25, 27).

Systematic documentation of AEs is crucial, as cancer treatment, inactivity, and late effects of multimodal therapy may increase the risk of AEs during exercise due to pain (28, 29), balance disturbances (30, 31), functional impairments (32), bone density loss (33), muscle weakness, and neurological impairments (29, 30, 34). Despite these concerns, comprehensive data on the frequency, consequences, outcomes, and contextual conditions of AEs during supervised exercise in children and adolescents with cancer remain limited, particularly with regard to therapy phase, diagnosis, and age. This gap restricts reliable conclusions on exercise safety in this population. A retrospective analysis conducted in Germany in 2020 addressed this issue by documenting AEs during 35,110 supervised regular exercise sessions from 24 centers (22). The study reported 983 grade 1 AEs, with muscle soreness being the most frequent (43%), and a very low incidence of grade 2 and 3 AEs (0.017%).

Some recommendations for action to reduce AEs in pediatric exercise oncology are already addressed in the German S2k guidelines on supervised regular exercise sessions in pediatric oncology developed by the Association of the Medical Societies in Germany (AWMF) (35, 36). These guidelines provide general specifications regarding blood count thresholds, exercise expert-to-patient ratios, and the overall framework for supervised exercise. However, specific instructions for preventing AEs have not yet been incorporated, primarily due to limited empirical data. Similarly, the international Pediatric Oncology Exercise Guidelines (iPOEG) refer to AEs, but do not include concrete, evidence-or consensus-based recommendations (3).

Accordingly, there is a clear need for systematic and prospective investigation of AEs during supervised exercise sessions with children and adolescents during and after cancer treatment. The aims of the present study are therefore:

(1) to prospectively describe incidence and characteristics of AEs during exercise sessions in pediatric oncology,
(2) to examine explore potential trends in AE grade and AE triggers, and
(3) to derive practical recommendations to reduce the risk for AEs during supervised exercise sessions in this population, informed by the empirical findings.

## 2 Materials and methods

**Figure 1:**
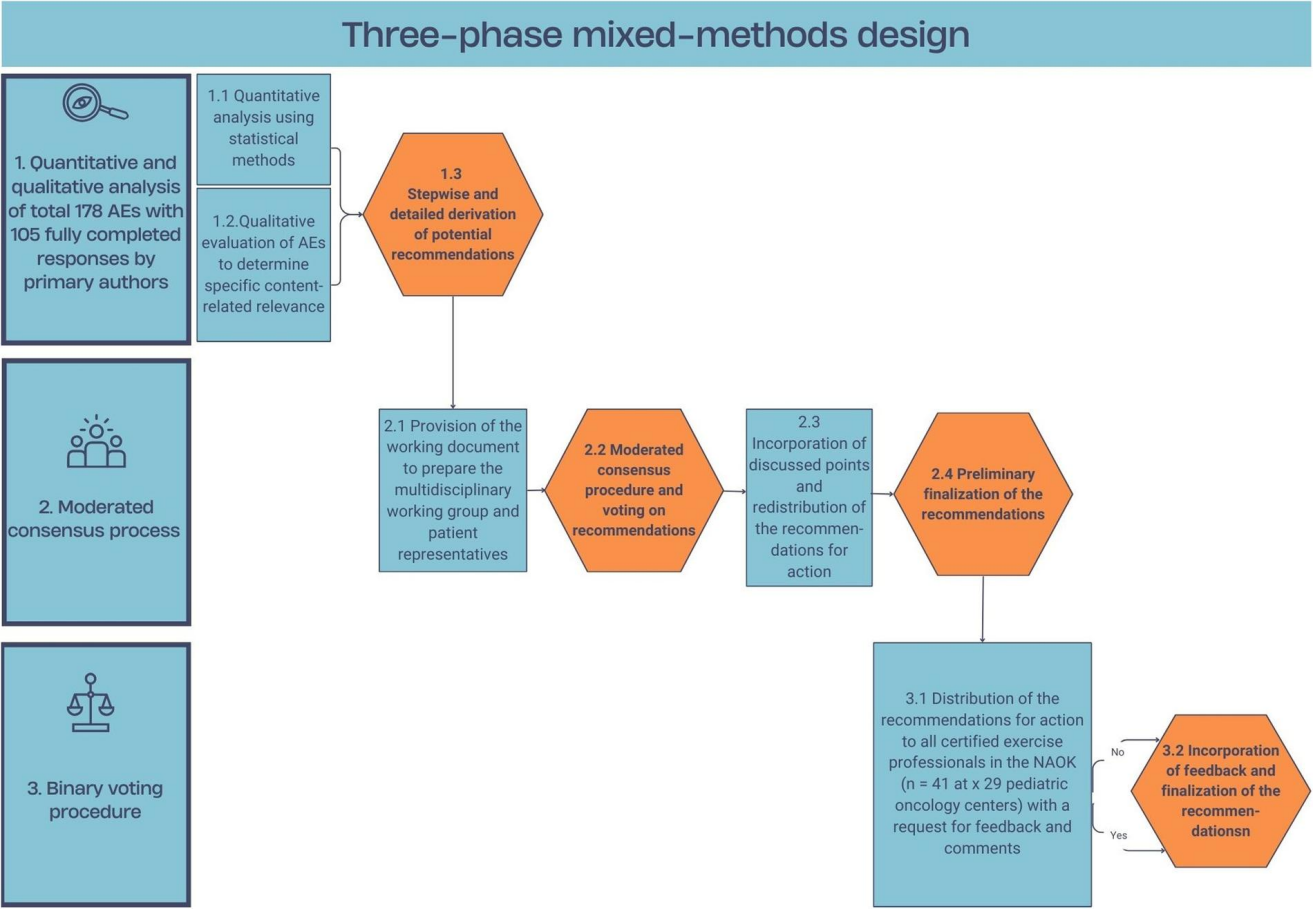
Three-phase mixed-methods design for characterizing adverse events during exercise sessions, and developing practical, evidence-based recommendations for action to reduce adverse events. AE, adverse event; n, number of participants; NAOK, Network ActiveOncoKids.

This prospective, multicenter observational study (January 2021–December 2024) used a structured, guideline-based assessment framework to prospectively and comprehensively collect AEs during supervised regular exercise sessions, helping to reduce the risk of reporting bias and enabling characterization of the AEs and their settings. As illustrated in Figure 1, a three-phase mixed-methods design was applied, combining a descriptive analysis of the AEs with a systematic method for developing practical, empirically informed recommendations to reduce AEs.

The responsible ethics committee (Ethics Committee of the Medical Faculty, University of Duisburg-Essen (Germany) confirmed in advance that no formal application was required because of the study's anonymized and non-interventional design.

### 2.1 Study population

All participating acute-treatment hospitals were affiliated with the German Society for Pediatric Oncology and Hematology (GPOH) and were part of the nationwide Network ActiveOncoKids (NAOK) initiative in Germany. The study involved five acute-care pediatric oncology centers and one rehabilitation facility, each contributing data for at least 12 months from usual care exercise programs. Eligible participants were children aged 3 years and older, adolescents, and young adults up to 21 years of age who had been diagnosed with cancer and who participated in supervised exercise programs during the acute, maintenance, or post-treatment phases at the respective centers. Exercise programs were generally offered to all patients and tailored to the patient's current health status, treatment phase, and clinical condition in consultation with the treating medical team (35, 36). Due to data protection regulations, detailed information on individual diagnoses and treatment regimens was not available for the present analysis. However, participating centers covered a broad spectrum of pediatric oncology diagnoses, including leukemia, central nervous system tumors, lymphomas, and solid tumors.

No minimum fitness level was required for participation in the exercise sessions or the present study (see Inclusion and Exclusion Criteria), as the exercise program was tailored to each participant's fitness level. Tailoring was based on participants' age, individual goals, prior exercise experience, personal preferences, overall health status, and day-to-day condition. Accordingly, key training parameters such as exercise type, intensity, duration, and progression were adjusted. When necessary, programs were further modified in consultation with the interdisciplinary treatment team, including specialists from cardiology, nursing, physiotherapy, pulmonology, and psychology.

### 2.2 Inclusion and exclusion criteria

In accordance with current guidelines on exercise and physical activity in pediatric oncology (35, 36), no minimum fitness level was required for participation in the exercise sessions or for inclusion in the present study, as the program was individually tailored to each participant's physical condition. These guidelines specify that no absolute exclusion criteria exist, and that participation is determined by relative contraindications depending on the patient's current clinical status. Even very low-intensity interventions, such as progressive muscle relaxation in patients with poor general condition, were considered part of exercise therapy according to this definition of movement-based programs.

### 2.3 Supervised exercise

In addition to physiotherapy, all sites offered supervised regular exercise sessions. The goal of these interventions was to reduce therapy-associated and inactivity-related side effects, prevent late and long-term effects of medical treatment, and leverage the positive effects of physical activity on participants' physical and mental health. Exercise parameters were adjusted to each patient's current condition. They targeted core motor components such as strength, endurance, flexibility, coordination, speed, and mental training, which were combined and adjusted to the participant's exercise capacity. Activities ranged from light (e.g., reaction games for coordination training) to moderate (e.g., muscle strengthening with small hand weights while sitting or standing, or games involving throwing or returning a ball) to more intensive exercises (e.g., interval-based endurance training on an ergometer). All supervised exercise sessions were conducted by qualified exercise experts with specific expertise in pediatric oncology. While supervision by trained professionals was ensured across all centers, exercise professional-to-patient ratios during individual sessions were not systematically documented. Sessions were held 3 to 5 times a week, lasting at least 15 min and typically 30 to 60 min. Supervision was defined as continuous instruction and monitoring by an exercise expert, either in person or via real-time video communication. In the online setting, this enabled immediate feedback, correction of exercise technique, and individualized adjustment of exercises to ensure safety and proper execution. For online training, exercises were selected conservatively to minimize fall risk, and only participants deemed capable of safely performing the exercises at home were included. In addition, an adult was required to be present in the household during all online sessions. The number of exercise sessions conducted at each participating hospital was documented, reported every 6 months, and submitted to the study office.

### 2.4 Definition and recording of AEs

Consistent with the definition used by Gauss et al. (22), an AE was defined as any new physical or psychological symptom occurring for the first time during a supervised regular exercise session or worsening over the course of that session. This definition implies, for example, that pain following a fall onto the knee is judged as a new-onset AE, whereas symptoms already present before the session, such as nausea, are considered AEs when they worsen during the exercise session, for instance progressing to vomiting.

### 2.5 Documentation

AEs were reported by the responsible exercise expert to the study coordinator via telephone using a guideline-based interview that included both free-text fields and predefined answer categories. For some variables, only a single response option was available (e.g., location), whereas for others multiple responses could be selected (e.g., affected body parts).

The data collection process was iteratively refined over the course of the study within an agile framework. In particular, contextual variables such as consequences of AEs and their duration were introduced at later stages and were therefore not available for earlier cases. In some instances, missing information could be retrospectively derived from free-text entries; however, this was not consistently possible. Thus, missingness reflects the stepwise expansion of the documentation system rather than random data loss.

All information was entered in the REDCap digital database (Research Electronic Data Capture, developed by Vanderbilt University, Nashville, TN, USA; Version 15.9.1), hosted on a secure university hospital server (Essen University Hospital), and based on predefined questions covering four categories (Table 1) (entry template: https://www.activeoncokids.org/wp-content/uploads/2025/11/AERegistry_AERegistry-2.pdf or Supplementary Material File S1). Participants' fitness level was also assessed by the exercise experts and categorized using a three-level rating system (moderate, average, good), based on comparison with age- and sex-specific expected fitness levels. Definitions of AE triggers and AE types were specified *a priori* and are provided in Supplementary Material Table S1.

**Table 1:** Recorded variables for each adverse event during supervised exercise sessions in childhood cancer.

Category*	Recorded variables
Basic information	Date; AE type**; AE trigger**; affected body parts**.
Judgment	CTCAE grading, exercise related (yes vs. no), new onset and exercise-related.
Consequences and outcomes (duration & intensity)	Hospitalization; increased care needs; medication administration; fear and uncertainty** (among the treatment team, the affected individuals, the parents, and the exercise experts); refusal of further exercise offers; application of the RICE rule; temporary suspension of session; termination of session; programmatic adaptation* (communication strategy, equipment, exercise selection, intensity, location); life-threatening consequences or death.
Background and setting	Therapy phase; group size; patient age; time point; location; primary motor form*; fitness level.

AE, adverse event; CTCAE, common terminology criteria for AE.

* Full category lists for all variables that were not recorded as free text are provided in Supplementary Material, File S1 or under the link: https://github.com/smeisegeier/sport-adverse-events/tree/v1.0.

** The questions underlying these variables were designed as multiple-response items.

### 2.6 Grading of adverse events

To ensure systematic grading of the reported AEs, a multilevel evaluation procedure based on the CTCAE, version 5.0 (37, 38) was conducted every 6 months by a multidisciplinary expert group comprising experts in medicine (CS), epidemiology (CSR), and pediatric exercise oncology (TS, HS, MG, GG), together with project partners. In the first step, each AE was independently and confidentially graded using a five-point scale (grades 1 to 5) (38), which had been preadapted to the context of supervised exercise sessions. The decisive criterion for grading was the objectively medical or subjectively perceived consequence for the affected individual (Supplementary Table S2):

- Grade 1 Interruption of the exercise with content modification,
- Grade 2: Medical intervention [e.g., first aid following the “**R**est, **I**ce, **C**ompression, and **E**levation (RICE)” rule, provided it was administered to treat an actual injury and not solely for teaching purposes or to provide comfort],
- Grade 3: Medically relevant consequences (e.g., surgical procedures or functional impairments affecting daily activities),
- Grade 4: Life-threatening measures.
- Grade 5: Death.

In cases of disagreement in the initial assessments, a moderated consensus process was used until consensus was reached. To this end, two online meetings were held annually, during which unclear cases were discussed, and final grades were agreed on.

Exercise experts judged each AE as exercise-related or not, and whether it represented a new-onset symptom or a worsening of a pre-existing condition This assessment was performed in close consultation with the treating medical team at each center. AEs were considered exercise-related if they were judged unlikely to have occurred or worsened in the absence of physical activity (e.g., at rest). This pragmatic approach was applied across centers but relied on clinical judgment rather than predefined decision rules or formal standardization. In cases of ambiguity, particularly for symptoms such as nausea, dizziness, or pain, classification was based on the temporal relationship to exercise, symptom progression during the session, and overall clinical context.

### 2.7 Phase 1: development of recommendations by primary authors

Phase 1 consisted of two steps resulting in establishing data-driven recommendations (Figure 1). First, a quantitative analysis was performed (see Statistical Analysis section). Second, the primary authors (GG, MG) qualitatively and contextually screened all free-text responses using qualitative content analysis (39) to systematically identify specific irregularities, cases of precedence, and particular learning cases that could inform the development of evidence-based recommendations for action to reduce AEs.

### 2.8 Phase 2: finalization of recommendations by a multidisciplinary expert group

Phase 2 comprised four steps (Figure 1). The aim was to revise the evidence-based and practice-oriented draft of the recommendations from phase 1 in detail. For this purpose, a multidisciplinary working group consisting of eight experts from the fields of medicine, sports science, physical therapy, epidemiology, and nursing, as well as patient representatives, was convened (MG, CSR, CS, TS, HS, GG, MD, MM). The group integrated professional perspectives from the involved disciplines. All participants were provided with comprehensive and transparent information on the development process of the recommendations, including their rationale and wording, in order to ensure traceability and plausibility. The complete documentation is available from the authors upon request. Subsequently, the recommendations for action to reduce AEs were discussed in a 120-minute consensus meeting (40). Following an initial revision, a written round of comments was conducted via email before the final version was approved by all members.

### 2.9 Phase 3: external validation using voting by practice-based experts

Phase 3 comprised two steps to externally validate the eleven recommendations for action to reduce AEs (Figure 1) (41). For this purpose, all exercise experts working in German-speaking pediatric oncology centers who had completed GPOH-certified BOP training [“Bewegungstherapeutische InterventiOnen der Pädiatrischen Onkologie” (Exercise Interventions in Pediatric Oncology)] were invited to provide their assessment. External validation was conducted using a standardized online survey via the LimeSurvey application (LimeSurvey GmbH, Hamburg, Germany; Version 6.10.3 + 250203). Participants were provided with background information on the development process of the recommendations. They were then asked to approve or reject the recommendations and were also given the opportunity to provide comments.

### 2.10 Statistical analysis

To standardize exercise units in relation to the occurrence of AEs, each child's and adolescent's participation in an individual or group exercise session lasting at least 15 min was counted as one exercise unit. Accordingly, ten children participating in individual sessions corresponded to ten exercise units, while a group session involving 15 adolescents corresponded to 15 exercise units. Longer interventions (e.g., activity days or multi-day programs) were likewise counted per participating child/adolescent and per session, such that a six-day skiing program with eight children completing two sessions per day resulted in a total of 96 exercise units (8 × 6 × 2). The number of sessions stratified by format (e.g., individual vs. group) was not systematically recorded across centers.

Descriptive analysis of the 178 recorded AEs was conducted using Python (Python scripts were developed by SM), and the aggregated dataset was made available in tabular form on Github (https://github.com/smeisegeier/sport-adverse-events/tree/v1.0) (Supplementary Dataset 1). Multiple responses were documented sequentially and were not assigned to mutually exclusive categories.

Complete information was available for 105 recorded AEs. Missing data most frequently affected contextual variables, particularly consequences of AEs, their duration, and session-specific characteristics. Within this subgroup, we investigated differences in AE grade and trigger in relation to AE type, therapy phase, group size, exercise relatedness, impact on session continuation, age, location, and motor performance using univariate group comparisons. CTCAE grades 2 and 3 were merged into a single moderate-to-severe category. Group differences were assessed using chi-square tests in IBM SPSS Statistics (IBM Corp., Armonk, NY, USA; Version 31.0.1.0), with a significance level of α = 0.05. As assumptions for chi-square testing were frequently violated, *p* values were calculated using Fisher's exact test with Monte Carlo simulation. Multiple responses were treated as independent categories; for example, AEs attributed to both medical therapy and physical (over)exertion were counted as an additional combined category. Given the exploratory nature of the analyses, no formal correction for multiple testing was applied. Accordingly, reported *p*-values should be interpreted as descriptive and hypothesis-generating rather than confirmatory, and the risk of type I error should be considered. Proportions are reported with 95% confidence intervals (CI). All analyses were exploratory and descriptive in nature.

## 3 Results

### 3.1 Incidence of adverse events

During the 3-year observation period, 178 AEs were documented across a total of 74,083 exercise units of supervised exercise sessions. Of these AEs, 133 (75%) were judged as grade 1, 42 (23%) as grade 2, and three (2%) as grade 3. The overall incidence rate was 240 AEs per 100,000 exercise units (0.2%), corresponding to one AE per 416 exercise units. On average, one grade 1 AE approximately every 561 exercise units, one grade 2 AE every 1,723 exercise units, and one grade 3 AE every 24,694 exercise units. Overall, a grade 2 or 3 AE occurred every 1,611 exercise units. Of the 178 AEs documented during the exercise sessions, 146 (82%) were judged as new-onset AEs, indicating that no symptoms had been present prior to the respective exercise unit. In 32 AEs (18%), pre-existing symptoms were present and worsened during the session. Among the 178 AEs, 151 (85%) were judged as exercise-related and were considered unlikely to have occurred in the absence of the exercise session. In contrast, 27 AEs were judged as non–exercise-related AEs and were considered likely to have occurred independently of physical activity, for example while watching a movie. Overall, 132 of the 178 documented AEs (74%) met both criteria simultaneously, namely new-onset and exercise-relatedness, indicating that the AE occurred during the exercise session and did not represent a worsening of any pre-existing symptoms (Table 2).

**Table 2:** Distribution of adverse events by CTCAE grading, onset status, and exercise relatedness during supervised exercise sessions.

Parameter	Grade 1	Grade 2	Grade 3	Total
	*N* (%)	*N* (%)	*N* (%)	*N* (%)
CTCAE grading	133 (75%)	42 (23%)	3 (2%)	178 (100%)
Onset status	132 (74%)	43 (24%)	3 (2%)	178 (100%)
New onset	109 (61%)	35 (20%)	2 (1%)	146 (82%)
Pre-existing and aggravated	23 (13%)	8 (5%)	1 (1%)	32 (18%)
Exercise-related	113 (63%)	36 (20%)	2 (1%)	151 (85%)
New onset + exercise- related	97 (74%)	33 (25%)	2 (2%)	132 (71%)

Remarks: The total number of events varies due to missing information in some of the variables.

AE, adverse event; CTCAE, common terminology criteria for AE; N, total number; n, number of participants.

### 3.2 Characteristics of adverse events

Overall, AEs were predominantly mild and observed across all age groups, therapy phases, and fitness levels. Higher-grade AEs were rare and were observed more frequently in specific age groups, therapy phases, and fitness levels. Grade 3 AEs were observed only in patients aged ≥15 years. In the >18-year age group, grade 2–3 AEs were descriptively more frequent than grade 1 AEs (5 vs. 3 cases), whereas grade 1 AEs predominated in all other age groups. During acute cancer treatment, the ratio of grade 2–3 to grade 1 AEs was 27:120, whereas during exercise in the post-treatment phase it was 16:9. All grade 3 AEs occurred in participants judged as having a “moderate” fitness level. Across all fitness levels, grade 1 AEs were the most frequently documented category (Table 3).

**Table 3:** Distribution of adverse events by patient age, therapy phase, and fitness level during supervised exercise sessions.

Parameter	Grade 1	Grade 2	Grade 3	Total
	*N* (%)	*N* (%)	*N* (%)	*N* (%)
Age (*n* = 133)				
2–5 years	13 (10%)	9 (7%)	0	22 (17%)
6–9 years	34 (26%)	6 (5%)	0	40 (30%)
10–14 years	30 (23%)	9 (7%)	0	39 (29%)
15–18 years	17 (13%)	6 (5%)	1 (1%)	24 (18%)
>18 years	3 (2%)	3 (2%)	2 (2%)	8 (6%)
Therapy phase (*n* = 178)				
Acute treatment	119 (67%)	27 (15%)	1 (1%)	147 (83%)
Maintenance	4 (2%)	2 (1%)	0	6 (3%)
Post-treatment	9 (5%)	14 (8%)	2 (1%)	25 (14%)
Fitness level (*n* = 124)				
Moderate	53 (43%)	17 (14%)	3 (2%)	73 (59%)
Average	26 (21%)	10 (8%)	0	36 (29%)
Good	10 (8%)	2 (2%)	0	12 (10%)
Unknown	3 (2%)	0	0	3 (2%)

Remarks: The total number of events varies due to missing information in some of the variables.

CTCAE, common terminology criteria for AE; N, total number; n, number of participants.

As summarized in Table 4, AEs occurred across a broad range of AE types, AE triggers, and affected body parts. The most frequent AE type combinations were soft-tissue or tissue injuries accompanied by pain (*n* = 13; 7%) and superficial injuries with pain (*n* = 9; 5%), with pain being reported across all severity grades. Regarding AE triggers, the most frequently documented causes were physical (over)exertion in combination with the consequences of medical treatment (*n* = 64; 36%), followed by fall-related incidents due to coordination problems (*n* = 10; 6%). For fall-related incidents, the ratio of grade 2–3 to grade 1 AEs was 20:21. With respect to affected body parts, grade 2–3 AEs were as frequent as grade 1 AEs in cases involving head injuries, whereas grade 1 AEs predominated in all other body parts.

**Table 4:** Distribution of adverse event types, triggers, and affected body parts by CTCAE grade.

Parameter	Grade 1	Grade 2	Grade 3	Total
	*N* (%)	*N* (%)	*N* (%)	*N* (%)
AE type (*n* = 178)*				
Pain	62 (35%)	30 (17%)	2 (1%)	94 (53%)
Nausea/vomiting	31 (17%)	4 (2%)	0	35 (20%)
Circulatory problems	28 (16%)	2 (1%)	0	30 (17%)
Soft-tissue injury	7 (4%)	14 (8%)	0	21 (12%)
Superficial injuries	7 (4%)	8 (4%)	0	15 (8%)
Psychological stress reaction	6 (3%)	1 (1%)	0	7 (4%)
Muscle soreness	4 (2%)	2 (1%)	0	6 (3%)
Physical (over)exertion	4 (2%)	0	0	4 (2%)
Coughing fit	4 (2%)	0	0	4 (2%)
Itching	3 (2%)	1 (1%)	0	4 (2%)
Bone injuries	0	0	2 (1%)	2 (1%)
Enuresis	2 (1%)	0	0	2 (1%)
Nosebleed	1 (1%)	1 (1%)	0	2 (1%)
Spontaneous painful bowel movement	2 (1%)	0	0	2 (1%)
Muscle cramps	1 (1%)	0	0	1 (1%)
AE trigger (*n* = 178)*				
Physical (over)exertion	93 (52%)	17 (10%)	2 (1%)	112 (63%)
Medical treatment	67 (38%)	11 (6%)	0	78 (44%)
Fall-related incident	21 (12%)	19 (11%)	1 (1%)	41 (23%)
Coordination problems	14 (8%)	8 (4%)	1 (1%)	23 (13%)
Psychological stress	11 (6%)	2 (1%)	0	13 (7%)
Collision	7 (4%)	2 (1%)	0	9 (5%)
Environmental conditions	5 (3%)	2 (1%)	0	7 (4%)
Other	1 (1%)	0	0	1 (1%)
Affected body parts (*n* = 177)*				
Internal organ	59 (33%)	6 (3%)	0	65 (37%)
Lower extremities	30 (17%)	11 (6%)	2 (1%)	43 (24%)
Head	10 (6%)	11 (6%)	0	21 (12%)
Abdomen	7 (4%)	4 (2%)	0	11 (6%)
Upper extremities	5 (3%)	6 (3%)	0	11 (6%)
Back	6 (3%)	3 (2%)	0	9 (5%)
Full body	6 (3%)	2 (1%)	1 (1%)	9 (5%)
Buttocks	5 (3%)	2 (1%)	0	7 (4%)
Chest	4 (2%)	1 (1%)	0	5 (3%)
Bowel	2 (1%)	0	0	2 (1%)
Coccyx	2 (1%)	0	0	2 (1%)
Genital area	0	1 (1%)	0	1 (1%)

Remarks: The total number of events varies due to missing information in some of the variables.

AE, adverse event; CTCAE, common terminology criteria for AE; N, total number; n, number of participants.

* Multiple responses possible.

With regard to AE characteristics, a total of 81 AEs (49%) occurred during coordination-related activities. All grade 3 AEs were associated with coordination exercises (*n* = 3) and/or strength exercises (*n* = 1). With respect to the location of occurrence, the ratio of grade 2–3 to grade 1 AEs was 21:41 in the exercise room, 8:33 in the patient's room, and 7:34 in the hospital corridor. Two grade 3 AEs occurred in the exercise room and one in an outdoor area. During testing procedures, four grade 1 AEs were documented, whereas during telemedicine sessions, five grade 1 and two grade 2 AEs were recorded. During individual exercise sessions, the ratio of grade 2–3 to grade 1 AEs was 33:127, while in group sessions it was 7:1. Regarding timing, information was available for 131 AEs, of which 44% occurred during the first half of the exercise session, while grade 2 and 3 AEs were more frequent in the second half. No life-threatening or fatal AEs were observed (Table 5).

**Table 5:** Distribution of exercise-related conditions and session characteristics by CTCAE grade.

Parameter	Grade 1	Grade 2	Grade 3	Total
	*N* (%)	*N* (%)	*N* (%)	*N* (%)
Main motor skill (*n* = 165)*				
Coordination	59 (36%)	19 (12%)	3 (2%)	81 (49%)
Endurance	37 (22%)	11 (7%)	0	48 (29%)
Flexibility	21 (13%)	3 (2%)	0	24 (15%)
Multimodal	19 (12%)	5 (3%)	0	24 (15%)
Relaxation	2 (1%)	0	0	2 (1%)
Speed	5 (3%)	5 (3%)	0	10 (6%)
Strength	27 (16%)	12 (7%)	1 (1%)	40 (24%)
During diagnostic testing (*n* = 177)	4 (2%)	0	0	4 (2%)
Group size (*n* = 174)				
Individual	126 (72%)	32 (18%)	2 (1%)	160 (92%)
Group (2–5)	1 (1%)	1 (1%)	0	2 (1%)
Group (5–10)	1 (1%)	4 (2%)	1 (1%)	6 (3%)
Group (>10)	0	6 (3%)	0	6 (3%)
Termination of session (*n* = 131)				
First half	50 (38%)	6 (5%)	2 (2%)	58 (44%)
Second half	46 (35%)	26 (20%)	1 (1%)	73 (56%)
Location (*n* = 162)				
Exercise room	42 (26%)	19 (12%)	2 (1%)	63 (39%)
Hospital corridor	34 (21%)	7 (4%)	–	41 (25%)
Outdoors	6 (4%)	4 (3%)	1 (1%)	11 (7%)
Patient room	32 (19,8)	8 (5%)	–	40 (25%)

Remarks: The total number of events varies due to missing information in some of the variables.

CTCAE, common terminology criteria for adverse events; N, total number; n, number of participants.

* Multiple responses possible.

### 3.3 Clinical and individual consequences of adverse events

With respect to the clinical and individual consequences of AEs, information on whether an AE led to termination of the exercise session was available for 162 documented AEs. Of these, 93 (57%) resulted in termination of the session. Information on the application of the RICE rule was available for 178 AEs and was required in 18 cases (10%). Data on programmatic adaptations were available for 178 AEs, of which 41 (23%) necessitated such adaptations, most commonly involving modifications to exercise content and exercise intensity. These were partly temporary and limited to a single session (e.g., avoiding upper-limb exercises on a day with arm pain and focusing on lower-limb training), whereas in other cases they were permanent (e.g., permanently avoiding ball games with hard balls following a painful impact to a Broviac catheter). Information on emotional reactions in terms of fear or uncertainty was available for 176 AEs and was documented in 57 cases (32%), predominantly among the affected patients themselves, with concurrent involvement of multiple parties in some cases. Across affected individuals, parents, and the treatment team, these reactions occurred most frequently in response to grade 1 AEs, were less common for grade 2 AEs, and rare for grade 3 AEs. In contrast, exercise experts reported fear or uncertainty slightly more often in response to grade 2 AEs than to grade 1 AEs (Table 6).

**Table 6:** Distribution of consequences and outcomes following adverse events by CTCAE grade.

Parameter	Grade 1	Grade 2	Grade 3	Total
	*N* (%)	*N* (%)	*N* (%)	*N* (%)
Hospitalization (*n* = 178)	1 (1%)**	0	1 (1%)	2 (1%)
Increased care needs (*n* = 178)	1 (1%)**	0	3 (2%)	4 (2%)
Medication administration (*n* = 173)	0	2 (1%)	3 (2%)	5 (3%)
Fear and uncertainty (*n* = 176)				
YES PRESENT*	34 (19%)	21 (12%)	2 (1%)	57 (32%)
Among the treatment team	9 (5%)	1 (1%)	2 (1%)	12 (7%)
Among the affected individuals	33 (19%)	19 (11%)	2 (1%)	54 (31%)
Among the parents	2 (1%)	3 (2%)	1 (1%)	6 (3%)
Among the exercise experts	3 (2%)	4 (2%)	1 (1%)	8 (5%)
RICE rule (*n* = 178)	2 (1%)	14 (8%)	2 (1%)	18 (10%)
Impact on session continuation (*n* = 162)				
Temporary suspension of session	53 (33%)	15 (9%)	1 (1%)	69 (43%)
Termination of session	69 (42%)	22 (14%)	2 (1%)	93 (57%)
Adaptations (*n* = 178)*				
YES PRESENT	37 (21%)	3 (2%)	0	40 (23%)
Communication strategy	3 (2%)	0	0	3 (2%)
Equipment	2 (1%)	1 (1%)	0	3 (2%)
Exercise selection	27 (15%)	3 (2%)	0	30 (17%)
Intensity	17 (10%)	1 (1%)	0	18 (10%)
Location	2 (1%)	0	0	2 (1%)
Life-threatening onsequences & death (*n* = 178)	0	0	0	0 (0%)

Remarks: The total number of events varies due to missing information in some of the variables.

CTCAE, common terminology criteria for AE; RICE-rule, rest, ice, compression, elevation; N, total number; n, number of participants.

* Multiple responses possible.

** It is assumed that these data originate from the very early phase of the project and were collected at a time when the guideline (Table X) was still being finalized. This case represents a non–exercise-related adverse event that occurred during an exercise session (associated with loss of the feeding tube and subsequent complications). Under normal circumstances, the criteria “hospitalization” and “increased care needs” would have classified this event as a Grade 2/3 adverse event.

### 3.4 Differences in grade and trigger by background variables

Based on 105 complete datasets (Supplementary Data S2), including only cases with complete information on all variables used in the comparative analyses, a detailed analysis was conducted to examine associations between AE grade and background characteristics. AE grade showed statistically significant associations with AE type, therapy phase, and group size but not with exercise relatedness, impact on session continuation, age, location, or motor performance (Table 7). With regard to AE type, Grade 1 AEs were predominantly general symptoms (52%, 95% CI: 41.0–62.7), whereas Grade 2–3 AEs were mainly injuries (46%, 95% CI: 29.5–64.2; *p* = 0.017), with different AE type distributions across severity categories. Concerning therapy phase, Grade 1 AEs predominated during acute cancer treatment (78%, 95% CI: 68.7–85.7), whereas in the post-treatment phase the relative proportion of Grade 2–3 AEs was higher (53%, 95% CI: 31.0–73.8; *p* = 0.012). Group size was also associated with AE severity (*p* = 0.042): individual sessions were predominantly associated with Grade 1 AEs (78%, 95% CI: 69.2–85.4), whereas group sessions were predominantly associated with Grade 2–3 AEs (88%, 95% CI: 52.9–97.8).

**Table 7:** Significant associations of AE grades and clinical and exercise-related parameters (*N* = 105).

Variable	Category	% (95% CI)	*p*-value (overall)	Direction of Association
AE type	Grade 1: general symptoms	52% (95% CI: 41.0–62.7)	0.017	Grade 1 → predominantly general symptoms; Grade 2/3 → predominantly injuries
	Grade 2: injuries	46% (95% CI: 29.5–64.2)		
Therapy phase	Acute treatment: Grade 1	78% (95% CI: 68.7–85.7)	0.012	Acute treatment → more Grade 1; post-treatment → relatively more Grade 2/3
	Acute treatment: Grade 2/3	22% (95% CI: 14.3–31.3)		
	Post-treatment: Grade 1	47% (95% CI: 26.2–69.0)		
	Post-treatment: Grade 2/3	53% (95% CI: 31.0–73.8)		
Group size	Individual sessions: Grade 1	78% (95% CI: 69.2–85.4)	0.042	Individual sessions → predominantly Grade 1 AEs; group sessions → predominantly Grade 2/3 AEs.
	Group sessions: Grade 2/3	88% (95% CI: 52.9–97.8)		
Exercise relatedness	–	–	0.731	No clear difference in AE grade distribution
Impact on session continuation	–	–	0.988	No clear difference in AE grade distribution
Age	–	–	0.142	No clear age-dependent pattern in AE grade
Location	–	–	0.315	No clear difference between locations
Motor performance	–	–	0.845	No clear pattern across motor performance types

Percentages are reported with 95% confidence intervals (CI).

CTCAE, common terminology criteria for adverse events (Grade 1 = mild, Grade 2 = moderate, Grade 3 = severe); *p* (MC), monte carlo–based *p*-value.

AE trigger showed statistically significant associations with AE type and impact on session continuation; no statistically significant associations were observed with exercise relatedness, therapy phase, group size, age, location, or motor performance (Table 8), although descriptive patterns were observed for several variables. Physical (over)exertion-related AEs were mainly associated with general symptoms (68%, 95% CI: 54.8–78.6), whereas collision- and fall-related AEs were predominantly associated with injuries (50%, 95% CI: 35.2–64.8) and pain (40%, 95% CI: 26.3–55.4; *p* < 0.001). Exercise relatedness differed by AE trigger: 75% (95% CI: 62.3–84.5) of physical (over)exertion-related AEs and 93% (95% CI: 80.1–97.4) of collision- and fall-related AEs were judged as exercise-related (*p* = 0.10). Impact on session continuation differed by AE trigger (*p* < 0.001): physical (over)exertion- and medical therapy–related AEs most frequently resulted in termination (70%, 95% CI: 56.7–80.1), whereas temporary suspension occurred in 30% (95% CI: 19.9–43.3); psychological strain was associated with termination in all observed cases (100%, 95% CI: 56.6–100.0). In contrast, collision- and fall-related AEs predominantly led to temporary suspension (73%, 95% CI: 57.2–83.9), with termination occurring less frequently (28%, 95% CI: 16.1–42.8). Descriptively, collision- and fall-related AEs were more frequent in younger children (2–9 years), whereas physical (over)exertion-related AEs predominated in older participants (10–18 years); physical (over)exertion-related AEs occurred with similar frequency in exercise rooms and patient rooms; and physical (over)exertion-related AEs were mainly observed during combination and coordination training, whereas collision- and fall-related AEs occurred most frequently during coordination training.

**Table 8:** Significant associations with Trigger and clinical and exercise-related parameters (*N* = 105).

Variable	Category	% (95% CI)	*p*-value (overall)	Direction of association
Exercise relatedness	Physical (over)exertion-related AEs: exercise-related	75% (95% CI: 62.3–84.5)	0.10	Collision/fall-related AEs → more often exercise-related
	Collision/fall-related incidents: exercise-related	93% (95% CI: 80.1–97.4)		
AE type	Physical (over)exertion: general symptoms	68% (95% CI: 54.8–78.6)	≤0.001	Physical (over)exertion → general symptoms; collision/fall → injuries/pain
	Collision/fall: injuries	50% (95% CI: 35.2–64.8)		
	Collision/fall: pain	40% (95% CI: 26.3–55.4)		
Impact on session continuation	Physical (over)exertion or medical therapy: suspension	30% (95% CI: 19.9–43.3)	≤0.001	Physical (over)exertion/medical therapy → termination; psychological strain → termination; collision/fall → predominantly suspension.
	Physical (over)exertion or medical therapy: termination	70% (95% CI: 56.7–80.1)		
	Psychological strain: termination	100% (95% CI: 56.6–100.0)		
	Collision/fall: termination-to-suspension ratio	73% (95% CI: 57.2–83.9)		
Therapy phase	Acute treatment: physical (over)exertion-related	60% (95% CI: 49.8–69.8)	0.062	Acute treatment → more physical (over)exertion; post-treatment → more collision/fall
	Post-treatment: collision/fall-related	71% (95% CI: 45.4–88.3)		
Group size	Group sessions: collision/fall-related incidents	100% (95% CI: 67.6–100.0)	0.066	Group sessions → collision/fall-related AEs
Age	Ages 2–9 years: collision/fall-related incidents	70% (95% CI 54.6–81.9)	0.166	Younger → collision/fall; older → physical (over)exertion
	Ages 10–18 years: physical (over)exertion-related	57% (95% CI: 44.1–69.2)		
Location	Exercise room: physical (over)exertion	38% (95% CI: 26.0–50.6)	0.063	Physical (over)exertion occurred similarly in exercise and patient rooms
	Patient room: physical (over)exertion	36% (95% CI: 24.5–48.8)		
Motor performance	Coordination training: collision/fall events	43% (95% CI: 24.5–63.5)	0.267	Physical (over)exertion → combination/coordination; collision/fall → coordination.
	Combination training: physical (over)exertion	55% (95% CI: 39.8–69.3)		
	Coordination training: physical (over)exertion	48% (95% CI: 28.3–67.6)		

CTCAE, common terminology criteria for adverse events (Grade 1 = mild, Grade 2 = moderate, Grade 3 = severe); p (MC), monte carlo–based *p*-value.

Percentages are reported with 95% confidence intervals (CI).

Supplementary Table S3 presents a categorical summary of the subgroup with complete data (*N* = 105) used for statistical analyses.

### 3.5 Recommendations for prevention of adverse events

Eleven recommendations for action to reduce AEs were developed through a multi-stage consensus process (Figure 1). A total of *n* = 37 of 41 individuals (90%) who had completed the BOP qualification and were officially listed participated in the binary voting procedure, excluding the four authors. Of these, *n* = 33 (80%) agreed with all recommendations; comments from those who disagreed are summarized in the following section.

The following section then presents the final recommendations, including a brief description of each recommendation, the underlying rationale, the corresponding voting results, a representative case study, and a summary of the comments provided by the respondents.

Recommendation 1 Brief description: Ensuring sound professional expertise grounded in current scientific evidence and guideline-based standards.

Recommendation: Given the high level of complexity in pediatric oncology, supervised regular exercise sessions should be designed and delivered by an exercise expert with specific qualifications in the following areas:

- Pediatric exercise oncology,
- Assessment of functional capacity and session monitoring (e.g., mobility, strength, endurance; Borg-based load regulation),
- Pediatric oncology–specific safety management (e.g., blood counts, red flags, infection risk, thrombocytopenia, fever, central venous catheter),
- Management of mobility restrictions, malalignment, and compensatory movement patterns,
- Exercise sessions for individuals with orthopedic and internal medicine-related conditions,
- Development of individualized training plans,
- Psychosocial aspects and motivational techniques,
- Counseling and communication skills.

Active involvement in scientific research and regular participation in continuing professional development are essential to ensure practice aligns with current scientific standards.

Rationale: The demonstrated positive effects of supervised regular exercise sessions underscore their clinical relevance in pediatric oncology. This complexity is reflected in the AE data. In the analyzed group (*n* = 105), group-based exercise sessions were exclusively associated with collision- and fall-related incidents, whereas physical (over)exertion predominated among adolescents aged 10–18 years, and collision- and fall-related incidents were most frequent among children aged 2–9 years. With respect to AE severity, individual exercise sessions were predominantly associated with Grade 1 AEs (78%; ratio Grade 1 to Grade 2–3 ≈ 3.6:1), whereas in group settings higher-grade AEs (Grade 2–3) predominated (88%; ratio Grade 2–3 to Grade 1 = 7:1). These findings highlight the need for individualized risk stratification and age-adapted exercise design, particularly with regard to group-based formats. Accordingly, supervised regular exercise requires experienced supervision and appropriate professional training.

Case study: Child aged 6–9 years, intensive therapy phase: pain in the tumor-affected leg following “ABCs of walking” instruction in the corridor.

Feedback from the binary voting procedure: 35 out of 37 reviewers endorsed the recommendation. Comments included: *For cases like this, specialized performance diagnostics or previous participation in scientific studies are not required. What matters is the ability to evaluate symptoms and apply evidence-based exercise guidelines to ensure safe and individualized program adjustment.*

Recommendation 2 Brief description: Preparing for potential physical incidents and managing fear or uncertainty with confidence and professionalism.

Recommendation: A specific emergency and intervention plan should be established (ideally as a standard operating procedure) in close collaboration with the multiprofessional team, taking local conditions into account and clearly defining responsibilities. A structured action plan should address the following key questions:

- Who should be notified in the event of an incident? (availability and awareness of relevant contact partners)
- What communication channels should be used for notification? (relevant telephone numbers stored in the official telephone or mobile phone)
- Which first aid measures may be performed independently? (e.g., use of first aid kit)

AEs or abnormalities with potential risk must be documented or communicated in a manner accessible to the multiprofessional team. Transparent, collegial, and reflective handling of AEs supports the ability to act effectively in challenging situations, promotes learning from critical incidents, and helps reduce long-term emotional stress for everyone involved. In addition, regular participation in cardiopulmonary resuscitation training and first aid refresher courses should be offered as part of routine clinical continuing professional development.

Rationale: In the overall dataset (*n* = 178), 80 responses indicated fear or uncertainty in one or more individuals. In 57 cases (32%), fear or uncertainty was explicitly reported as having occurred, with these reactions being expressed by the exercise experts themselves (5%), members of the treatment team (7%), patients (31%), and parents or other caregivers (3%). These findings demonstrate that AEs can have emotional as well as physical consequences. Regular exchange within the team members, for example through case-based discussions, may contribute to developing a confident and appropriate approach to AEs in critical situations.

Case study: Adult patient > 18 years, acute cancer treatment: After approximately 18 min of moderate exertion on a seated ergometer, the patient experienced dizziness and had to lie down.

Feedback from the binary voting procedure: 37 out of 37 reviewers endorsed the recommendation.

Recommendation 3 Brief description: Raising awareness of the importance of thorough preparation.

Recommendation: Exercise experts must always have access to patient records, be included in multidisciplinary meetings, and be provided with sufficient time for preparation. This enables them to plan, implement, and document exercise sessions in a manner that reduce risk. In cases of specific questions or uncertainties, the expertise of parents or other caregivers, as well as of the following professionals should be actively sought:

- Cardiologists
- Pediatric oncologists
- Members of the psychosocial team
- Hemato-oncology nurses/oncology nurses
- Physiotherapists
- Pulmonologists
- Pediatric orthopedic surgeons

Rationale: In 177 cases, documentation specified whether an AE was exercise-related. Of these, 85% were classified as exercise-related. In the analyzed group (*n* = 105), approximately half of the AEs were attributed to physical (over)exertion. This finding suggests that, in some cases, the kind and intensity of exercise sessions may not have been sufficiently tailored to the patient's individual health status. Accordingly, careful individualization of exercise intensity and close interprofessional coordination are essential.

Case study: Child aged 6–9 years, acute treatment phase: During coordination training, the patient suddenly developed severe stabbing pain in a previously irradiated part of the body.

Feedback from the binary voting procedure: 37 out of 37 reviewers endorsed the recommendation.

Recommendation 4 Brief description: Raising awareness of the mental situation of the target group.

Recommendation: Exercise experts should proactively address the emotional burden that arises from the sudden and severe impact of a life-threatening illness on patients. This involves obtaining relevant background information, consulting with psychosocial services, and maintaining a reflective, professional mindset characterized by empathy and sensitivity.

Rationale: In 13 cases (5%) of the total dataset (n = 178), an emotional stress reaction was documented as the trigger for an AE. Analysis of the subgroup (*n* = 105) further revealed that psychological stress reactions consistently led to immediate discontinuation of exercise sessions. In contrast, physical (over)exertion, collisions or falls, and reactions to medical treatment resulted in session termination in approximately two-thirds of cases, while temporary suspension occurred in about one-third of cases. These findings suggest that emotional factors may represent particularly consequential triggers for AEs.

Case study: Adolescent 15–18 years, intensive therapy phase: While in the corridor outside the stem cell transplant unit shortly before undergoing their own transplant, the patient experienced a psychological stress reaction and began to cry intensely.

Feedback from the binary voting procedure: 37 out of 37 reviewers endorsed the recommendation.

Recommendation 5 Brief description: Considering potential late and long-term effects.

Recommendation: During the post-treatment phase, structured and nuanced screening for potential delayed and long-term sequelae should be performed, tailored to the patient's individual medical history (e.g., fracture risk, cancer-related fatigue, neurological impairments, emotional stress). This process should be implemented sensitively to avoid causing unwarranted anxiety. Screening results should be incorporated directly into the selection and adaptation of exercise kind, equipment settings, and supervision ratios.

Rationale: Of the 178 documented AEs, 25 (14%) occurred in the post-treatment phase, with moderate-higher-grade AEs (grade 2–3) occurring 1.8 times more frequently than grade 1 AEs. In total, 46 grade 2–3 AEs were reported among 74,083 documented exercise units, corresponding to an incidence rate of ≈62 per 100,000 units. This represents a substantially lower risk than the general accident rate observed in school settings (5,670 per 100,000 students, source https://www.dguv.de/de/zahlen-fakten/schuelerunfallgeschehen/index.jsp). Analysis of the group (*N* = 105) shows significant differences between therapy phases. During post-treatment phase approximately twice as many grade 2–3 AEs occurred as grade 1 AEs, whereas during acute cancer treatment, the ratio was nearly 1:4 (grade 1 to grade 2–3). The main triggers during acute treatment were physical (over)exertion, while collision- and fall-related incidents predominated during the post-treatment phase. These findings emphasize the need to account for therapy phase–specific risk profiles when planning exercise sessions.

Case study: Child aged 6–9 years, post-treatment phase: The patient sustained a fatigue fracture after stopping abruptly while riding a scooter.

Feedback from the binary voting procedure: 36 out of 37 reviewers endorsed the recommendation. Comments included: *In posttreatment care, relevant medical information is typically obtained from parents or patients rather than directly from physicians, which frequently leads to delays in the flow of information.*

Recommendation 6 Brief description: Identifying potential risks of falls or collisions early on through environmental analysis.

Recommendation: A thorough inspection of the exercise environment is essential to ensure safety, particularly when therapy-related deficits are present, such as sensory impairments (e.g., vision or hearing loss), neurological deficits without sensory organ involvement, neuropathy, or muscle weakness and pain. Exercise experts must ensure that patients wear appropriate and functional clothing and that infusion lines or cables that could pose tripping or pulling hazards are properly managed. Additional environmental factors should also be considered, including floor texture, corners, window surfaces, and narrow corridors. Exercise planning should anticipate potential risks by providing clear instructions, implementing protective measures (e.g., use of mats, stable support options), and offering individualized assistance when gait or balance are compromised.

Rationale: Among the 178 AEs, fall-related incidents (23%), coordination problems (13%), and collisions (5%) collectively accounted for 41% of all AEs. In the analyzed group (*n* = 105), collision- and fall-related AEs were predominantly associated with pain and injuries, including soft-tissue and superficial injuries. These AEs were more frequently observed among higher-grade AEs 2–3 compared with grade 1 AEs. Qualitative case analyses further indicated that, in addition to the spatial setting, clothing and footwear may contribute to the risk of injury.

Case study: Adolescent aged 10–14 years, acute treatment: While playing a ball game, the patient struck their head on a window frame, sustaining a local soft-tissue contusion to the forehead and requiring platelet administration.

Feedback from the binary voting procedure: 37 out of 37 reviewers endorsed the recommendation.

Recommendation 7 Brief description: Providing patients with full attention during exercise sessions.

Recommendation: During exercise sessions, exercise experts should devote their full attention to the patient and communicate this clearly and empathetically at the beginning of each session. Opportunities for discussion with parents and family members should be proactively scheduled at other times, or the individuals should be referred to colleagues who are also responsible for the patient's care. When preparing exercise materials, therapists must ensure that children are continuously supervised and do not begin activities unsupervised. To minimize interruptions, a sign reading “Do not disturb—exercise session in progress” can be placed on the door. If the door is opened unexpectedly, the session should be briefly paused.

Rationale: Parents and caregivers of children/adolescents with cancer are often under considerable emotional strain. Qualitative analyses indicate that such interruptions can lead to safety-related distractions in the exercise location. Of the total dataset (*n* = 178), 162 cases included information on the exercise location. A substantial proportion of AEs (26%) occurred in the patient's room, where parents or caregivers are frequently present. However, these AEs were primarily grade 1 and 2 AEs, occurring at a ratio of approximately 1:4, with no grade 3 AEs recorded. The data suggest that parental presence in the patient's room may occasionally lead to distractions and mainly low-moderate-grade AEs.

Case study: Child aged 6–9 years, acute cancer treatment: During a balance exercise circuit, the patient unexpectedly changed direction, lost balance, and fell, sustaining a painful swelling on the back of the head. At the time of the fall, the exercise expert was engaged in conversation with the patient's mother.

Feedback from the binary voting procedure: 35 out of 37 reviewers endorsed the recommendation. Comments included: *Therapy sessions may be briefly interrupted if necessary but can be continued at the patient's request. Communication with parents may take place concurrently, provided the therapist's primary focus remains on the child.*

Recommendation 8 Brief description: Preventing physical and emotional overexertion through appropriate load management.

Recommendations: Exercise sessions should begin at a moderate intensity, guided by the Borg scale (11 to 13) (1). Exercise load should be monitored primarily through clinical observation and patient-reported exertion (e.g., Borg scale). Objective monitoring tools (e.g., pulse oximetry with integrated heart rate measurement) should be used when medically indicated, such as in children and adolescents with cardiopulmonary limitations, therapy-related respiratory impairment, or other relevant risk factors. Warning signals such as pallor, heavy sweating, breathlessness, dizziness, coordination disturbances, or noticeable behavior changes require adjustment of the exercise load. At the beginning of acute therapy, the focus should be on building a trusting relationship between the exercise expert and the patient. Exercise intensity and complexity should be kept low to prevent overexertion. In a second step, physical demands can be gradually increased in accordance with the principles of sports science and in close collaboration with the patient. Throughout, the exercise expert must remain mindful of the patient's individually reduced exercise capacity and their physical condition on that day.

Rationale: Patients do not always perceive or acknowledge their reduced exercise capacity, and it is not always externally apparent. Determining the appropriate level of exertion therefore requires careful, individualized assessment and continuous monitoring by the exercise experts. In the overall dataset (*n* = 178), the most frequent triggers of AEs—excluding outdoor activities—were physical (over)exertion alone or in combination with medical treatment (63%), independent of the exercise location. Group analysis (*n* = 105) showed that among adolescents aged 10–18 years, AEs most commonly occurred as a result of physical (over)exertion. In this age group, inactivity associated with adolescent lifestyle habits, combined with an impaired ability to accurately assess individual physical limits, may represent important contributing factors.

Case study: Adolescent aged 15–18 years, acute cancer treatment: During playful coordination exercises while standing on the stem cell transplant ward, the patient became dizzy and unwell. Shortly thereafter they briefly lost consciousness, collapsed, and were unresponsive for a short period.

Feedback from the binary voting procedure: 35 out of 37 reviewers endorsed the recommendation.

Recommendation 9 Brief description: Raising awareness for the appropriate use of materials.

Recommendations: Exercise experts must choose materials that are appropriate for the specific treatment situation, adhere to hygiene guidelines, and align with the patient's current state of health. Materials should be easy to handle, safe for use around intravenous infusion stands and cables, and must not come into contact with operated areas or medical access points.

Rationale: Qualitative analysis identified two AEs (1%) with material-related risks. These examples clearly demonstrate the importance of adapting the choice of materials to each patient's health condition.

Case study: Adolescent aged 10–14 years, acute cancer treatment: A small juggling ball fell onto a fresh surgical wound, causing pain.

Feedback from the binary voting procedure: 35 out of 37 reviewers endorsed the recommendation.

Recommendation 10 Brief description: Managing risks when using mobility aids and implementing preventive measures.

Recommendation: The use of mobility aids such as (sports) wheelchairs and forearm crutches must be assessed individually and tested under real-life conditions. Safe and reliable handling of the specific aid by the exercise expert is a prerequisite. Before use, all equipment or objects on which patients will sit must be evaluated for stability and functional suitability. For external exercise locations, it must be ensured that both the infrastructure and technical expertise allow for accessible participation by individuals using mobility aids.

Rationale: For patients with functional impairments of the lower extremities—such as during the preoperative phase following the diagnosis of a bone tumor, or as a result of surgical interventions or amputation—exercise sessions are often conducted using mobility aids like wheelchairs. While these aids are essential for maintaining participation and mobility, they may pose safety risks.

Case study: Adolescent (10–14 years), acute cancer treatment: During an exercise session incorporating playful, combative elements with the participant using a wheelchair, the wheelchair nearly tipped backward. The exercise expert intervened to stabilize it and unintentionally touched a highly sensitive area near the patient's recently amputated leg, causing pain and resulting in the patient crying.

Feedback from the binary voting procedure: 37 out of 37 reviewers endorsed the recommendation.

Recommendation 11 Brief description: Preventing coordination-related deficits and promoting of coordination skills.

Recommendation: Given the potential for coordination impairments resulting from neurotoxic chemotherapy agents, such as vinca alkaloids, platinum derivatives, and topoisomerase inhibitors, diverse coordination-focused exercise modalities should be continuously integrated into exercise sessions. Special emphasis should be placed on the lower extremities and on adolescents as a key target group. For practical implementation, exercise materials specifically developed for pediatric oncology should be used, such as topic-specific exercise brochures and instructional videos. A detailed overview of therapy-related adverse effects is provided in the AWMF S2k guidelines.

Rationale: In the overall dataset, 23 documented AEs (13%) were associated with coordination problems. In the analyzed group (*n* = 105), coordination-related motor content was frequently associated with collision- and fall-related AEs. Specifically, collision- and fall-related incidents occurred during coordination training in 43% of cases, whereas physical (over)exertion was primarily associated with combination training (55%) and coordination training (48%). The compromised exercise capacity caused by the disease and its treatment, along with various medications, can considerably impair coordination. Examples of these medications include neurotoxic chemotherapy agents such as:

- Vinca alkaloids (e.g., vincristine): the main cause of chemotherapy-induced peripheral neuropathy, leading to muscle weakness and coordination disturbances.
- Platinum derivatives (e.g., cisplatin, carboplatin): may cause gait instability, sensory deficits, and ataxia.
- Taxanes and oxaliplatin: associated with motor impairments, particularly with repeated administration.
- Topoisomerase inhibitors (e.g., etoposide, irinotecan), which can exacerbate coordination-restricting effects in association with combined therapies such as with platinum derivative.
- Anticonvulsants (e.g., levetiracetam, valproate): used for seizure prevention and treatment, but may cause sedation and delayed reaction times.
- Antiemetics (e.g., dimenhydrinate/Vomex): used to treat nausea and vomiting, often cause tiredness and reduced attentiveness.
- Analgesics (especially narcotics such as morphine or fentanyl): alleviate pain, but may reduce alertness, cause drowsiness, and impair motor control.

Additional medication classes that may significantly impair coordination include:

- Corticosteroids (e.g., dexamethasone, prednisolone): frequently cause proximal muscle weakness, myopathy, fatigue, mood changes, and impaired postural control.
- Benzodiazepines (e.g., midazolam, lorazepam, diazepam): used for procedural sedation or anxiety relief; may cause drowsiness, delayed reaction times, reduced coordination, and impaired balance.
- Beta-blockers (e.g., propranolol): may lead to dizziness, bradycardia, reduced exercise tolerance, and blunted perception of exertion.
- Sedating antihistamines (e.g., promethazine): can cause somnolence, impaired attention, and slowed reaction speed.

Neurotoxic chemotherapy, analgesics, anticonvulsants, sedatives, and corticosteroids are well-known to impair balance, coordination, alertness, and reaction time and may therefore substantially increase the risk of coordination-related AEs. A comprehensive overview of medication-related AEs and late- and long-term effects can be found in the AWMF S2k guidelines (35, 36).

Case study: Child aged 6–9 years, acute cancer treatment: During an exercise session to improve ball control, the patient lost balance when kicking the ball on one leg and fell on their buttocks, resulting in pain.

Feedback from the binary voting procedure: 35 out of 37 reviewers endorsed the recommendation.

## 4 Discussion

Supervised regular exercise sessions in pediatric oncology represent a promising supportive approach for symptom management and improvement of quality of life. The data in this study support previous findings demonstrating the overall safety of such supervised exercise sessions (22). No deaths, life-threatening complications, or delays in medical treatment occurred.

Most AEs documented during the exercise sessions were manageable through situational adaptation (e.g., modification of exercise type or intensity, shifting the focus to other body parts, taking breaks, or the terminating of individual sessions). Common AEs, such as pain, circulatory problems, or nausea/vomiting, may also occur independently of exercise as a result of the underlying disease or medical treatment (29).

Although very few AEs had long-term impacts, any additional strain represents a relevant concern for this vulnerable patient group. Patients already experience significant burdens due to chemotherapy (29, 30), radiotherapy, surgical procedures (31, 32), and cancer-related fatigue.

The results further indicate that the occurrence of AEs may be associated with therapy phase, group size, and age. The higher frequency of minor AEs (grade 1) in younger children may reflect more cautiously selected exercise-intensity parameters. In contrast, higher-grade AEs among adolescents may indicate age-related vulnerabilities or an overestimation of individual exercise capacity (42). The finding that larger groups were associated with more severe AEs suggests the potential importance of an appropriate expert-to-patient ratio in this setting. However, these results should be interpreted in light of the limited availability of detailed information on actual supervision ratios, as well as the lack of systematic data on the number of group sessions and corresponding exposure.

Physical (over)exertion, coordination problems, and fall-related incidents emerged as the primary triggers for AEs and session discontinuations, underscoring the need to further refine safety protocols and individualize exercise programming. In this context, the patient's ability to perceive and interpret physical (over)exertion is critical. This is particularly relevant in pediatric oncology, where bodily awareness, fatigue perception, and symptom interpretation may be limited or distorted by disease- or treatment-related effects. Exercise experts should therefore actively support patients in developing reliable self-monitoring skills. Guided experiences during exercise sessions, age-appropriate explanations, and the involvement of caregivers (especially for younger children) may help patients recognize effort, early warning signs, and safe exertion limits. Strengthening bodily awareness may contribute to enhanced safety and facilitates the transfer of self-regulation skills after acute cancer treatment into daily life, leisure activities, school and sport.

It is notable that anxiety- and insecurity-related reactions among exercise experts were most often associated with higher-grade AEs. This may be explained by the greater clinical relevance of such AEs, the need for immediate decision-making. These findings underscore the importance of adequate professional qualification and interdisciplinary collaboration.

To ensure safety in usual care exercise sessions, the recommendations for action to reduce AEs defined here should be understood as consensus-based and practice-informed guidance rather than evidence of proven efficacy Their implementation in clinical practice and integration into guidelines may be considered but should be further evaluated in prospective interventional studies.

### 4.1 Comparison with healthy children and adolescents

Several aspects of the “requirements for prevention” outlined by Nührenbörger et al. (43) for healthy individuals are also transferable to pediatric oncology. These include increasing training load gradually, avoidance of overload through appropriate recovery, promotion of neuromuscular control, and targeted development of strength and balance. Measures to enhance body awareness, adapt equipment, and consider motivational factors are also highly relevant. However, these principles cannot be applied directly to children and adolescents with cancer. Disease-specific physiological and mental factors, as well as treatment-related stressors, create fundamental differences that must be considered when designing and implementing exercise programs for this population.

### 4.2 Distinctive features of the recommendations

To address the distinct physical (44, 45) and emotional challenges (46, 47), as well as the specific contextual factors of pediatric oncology (35), the recommendations for action to reduce the risk of AEs were developed based on the empirical findings of this study and refined through multidisciplinary expert consensus. Assessment and judgment were conducted by multidisciplinary experts from pediatric oncology, nursing, sports science, and physiotherapy, as well as patient representatives.

A central aspect of these specific recommendations for action is the emphasis on close multiprofessional collaboration, supported by ongoing training for exercise experts and unrestricted access to patient-related information. Minimizing risks and reducing the likelihood of (over)exertion during supervised regular exercise sessions require an individualized, context-sensitive program design that reflects both the physical and mental burdens experienced by patients, as well as proactive identification of potential risk factors. This individualized and safety-aware perspective is consistent with existing literature in pediatric oncology. Review data suggest that exercise interventions are feasible and effective, while the overall evidence remains limited and methodologically heterogeneous (48). More recent intervention frameworks emphasize tailoring exercise prescription to age, treatment phase, functional capacity, and treatment-related impairments (49), while qualitative evidence further highlights the importance of patient-centered and context-sensitive physical activity approaches in this population (50).

### 4.3 Limitations

A substantial proportion of the recommendations are based on clinical experience, individual cases, and expert consensus and have not been formally evaluated within an interventional study design, which limits the available evidence base. Accordingly, these recommendations should be understood as practice-informed guidance rather than as evidence of proven efficacy. Methodologically, a major constraint is the high proportion of expected frequencies below 5, which violates the assumptions of chi-square testing. Although Monte Carlo *p* values were applied, the results should be interpreted with caution, as the small size of several subgroups based on completely recorded cases is reflected in wide 95% confidence intervals for many estimates, indicating considerable statistical uncertainty even where statistically significant associations were observed. Moreover, no correction for multiple testing was performed, increasing the risk of type 1 errors. In addition, all analyses were based on univariate group comparisons without adjustment for potential confounding variables such as age or exercise-related judgments. Therefore, the observed associations cannot be interpreted as independent effects. Furthermore, the analyses assume independence of observations, which may not fully account for potential clustering within individuals or centers, potentially leading to biased variance estimates and effect estimates. A multivariable approach, for example using logistic regression models, would in principle have been more appropriate to simultaneously account for multiple influencing factors and to reduce confounding bias. However, given the limited number of complete cases and the small and heterogeneous subgroup sizes, such models would likely have been statistically unstable and prone to overfitting. Further limitations arise from the dataset itself, including small overall case numbers, particularly within subgroups; incomplete population data; and the exclusive use of anonymized data, which precluded diagnosis-specific analyses. In addition, only 105 of 178 AEs were available for subgroup analyses due to missing data in variables that were introduced later during the study. As a formal analysis of missingness patterns across centers or AE severity was not feasible, a potential influence on the observed associations cannot be excluded. Furthermore, the use of aggregated exercise units as the denominator combines different types of exercise exposure and does not allow for setting-specific risk estimation. In addition, the total number of exercise units stratified by session format (e.g., individual vs. group sessions) was not available, limiting the interpretation of differences observed between formats, as these may reflect differences in exposure rather than true differences in safety profiles. Future studies should systematically collect these denominators to enable more precise comparisons across exercise settings. In addition, diagnosis-specific information and detailed data on supervision ratios (e.g., exercise expert-to-patient ratio) were not available due to anonymization and documentation constraints. These factors may influence the occurrence and characteristics of AEs and limit the interpretability of subgroup differences; therefore, the findings should be regarded as exploratory and hypothesis-generating, with limited generalizability. Future studies should aim to systematically collect these variables and validate the findings in larger, prospective studies using more robust multivariable methods.

### 4.4 Future directions

The beneficial impacts of supervised regular exercise sessions such as reducing therapy-associated and inactivity-related consequences and minimizing late- and long-term effects (3, 7, 11, 13, 51–53) are well documented. These advantages clearly outweigh potential risks. Nevertheless, certain specific risks exist and should be systematically assessed and proactively reduced through targeted prevention measures. In this context, existing evidence gaps highlight the need for a systematically maintained and detailed registry, together with regular revision and further specification of the corresponding recommendations. Based on the retrospective data collection conducted in 2021 (22) and the AE data from five pilot centers presented in this study, the future development of the AE project was initiated. This approach involves 17 pediatric oncology centers in Germany and Switzerland (DRKS00037046). It has received ethics approval, and will allow the collection of detailed medical data (e.g., diagnoses, cumulative chemotherapy doses). The long-term objective is to refine the recommendations for action to reduce AEs based on prospective data and to ensure their sustainable implementation in clinical practice.

## Data Availability

The datasets presented in this study can be found in online repositories. The names of the repository/repositories and accession number(s) can be found below: https://github.com/smeisegeier/sport-adverse-events/tree/v1.0.

## Supplementary material

Supplementary Table 1 Types of triggers contributing to adverse events – definitions and examples.

Supplementary Table 2 Adapted CTCAE criteria for grading the severity of adverse events (AEs).

Supplementary Table 3 Categorical summary of the group with completely recorded cases (N = 105) for statistical testing.

Supplementary Dataset 1 Raw data total (N = 178).

Supplementary Dataset 2 Raw dataset of complete data (N = 105).

Supplementary File 1 Data Collection Form (AE Registry).

## References

[1] Battanta N Lange K Kesting SV Marx-Berger D Heesen P Ober H Supervised physical activity interventions in children and adolescents with cancer undergoing treatment—a systematic review. Curr Oncol. (2025) 32:234. 10.3390/curroncol32040234 40277791 PMC12025492

[2] Caru M Dandekar S Schmitz KH. Physical activity and childhood cancer: present status and future directions. Am J Lifestyle Med. (2025):15598276251368342. 10.1177/15598276251368342 40852085 PMC12367733

[3] Wurz A Mclaughlin E Lategan C Chamorro Viña C Grimshaw SL Hamari L The international pediatric oncology exercise guidelines (iPOEG). Transl Behav Med. (2021) 11:1915–22. 10.1093/tbm/ibab028 34037786 PMC8604278

[4] Wellbrock M Borkhardt A Ronckers CM Spix C Grabow D Filbert AL Socioeconomic background and childhood cancer survival in Germany: a nationwide assessment based on data from the German childhood cancer registry. Int J Cancer. (2025) 157(11):2235–47. 10.1002/ijc.70042 40673348 PMC12496003

[5] Morales JS Valenzuela PL Rincon-Castanedo C Takken T Fiuza-Luces C Santos-Lozano A Exercise training in childhood cancer: a systematic review and meta-analysis of randomized controlled trials. Cancer Treat Rev. (2018) 70:154–67. 10.1016/j.ctrv.2018.08.012 30218787

[6] Caru M Levesque A Rao P Dandekar S Terry C Brown V A scoping review to map the evidence of physical activity interventions in post-treatment adolescent and young adult cancer survivors. Crit Rev Oncol Hematol. (2022) 171:103620. 10.1016/j.critrevonc.2022.103620 35104634

[7] Braam KI Van Der Torre P Takken T Veening MA Van Dulmen-Den Broeder E Kaspers GJ. Physical exercise training interventions for children and young adults during and after treatment for childhood cancer. Cochrane Database Syst Rev. (2016) 3:Cd008796. 10.1002/14651858.CD008796.pub3 27030386 PMC6464400

[8] Stössel S Neu MA Wingerter A Bloch W Zimmer P Paret C Benefits of exercise training for children and adolescents undergoing cancer treatment: results from the randomized controlled MUCKI trial. Front Pediatr. (2020) 8:243. 10.3389/fped.2020.00243 32582585 PMC7290004

[9] Fiuza-Luces C Padilla JR Soares-Miranda L Santana-Sosa E Quiroga JV Santos-Lozano A Exercise intervention in pediatric patients with solid tumors: the physical activity in pediatric cancer trial. Med Sci Sports Exerc. (2017) 49:223–30. 10.1249/MSS.0000000000001094 27631396

[10] Saultier P Vallet C Sotteau F Hamidou Z Gentet JC Barlogis V A randomized trial of physical activity in children and adolescents with cancer. Cancers. (2021) 13:121. 10.3390/cancers13010121 33401713 PMC7795208

[11] Benzing V Siegwart V Spitzhüttl J Schmid J Grotzer M Roebers CM Motor ability, physical self-concept and health-related quality of life in pediatric cancer survivors. Cancer Med. (2021) 10:1860–71. 10.1002/cam4.3750 33527768 PMC7940246

[12] Basteck S Guder WK Dirksen U Krombholz A Streitbürger A Reinhardt D Effects of an exercise intervention on gait function in young survivors of osteosarcoma with megaendoprosthesis of the lower extremity—results from the pilot randomized controlled trial proGAIT. Curr Oncol. (2022) 29:7754–67. 10.3390/curroncol29100613 36290890 PMC9599989

[13] Beller R Bennstein SB Götte M. Effects of exercise interventions on immune function in children and adolescents with cancer and HSCT recipients—a systematic review. Front Immunol. (2021) 12:746171. 10.3389/fimmu.2021.746171 34646274 PMC8504856

[14] Bucciarelli V Bianco F Bisaccia G Galanti K Arata A Ricci M Prevention of cardiotoxicity in childhood cancer survivors: in physical exercise, we trust. Curr Probl Cardiol. (2024) 49:102722. 10.1016/j.cpcardiol.2024.102722 38908726

[15] Morales JS Valenzuela PL Velázquez-Díaz D Castillo-García A Jiménez-Pavón D Lucia A Exercise and childhood cancer-A historical review. Cancers. (2021) 14:82. 10.3390/cancers14010082 35008246 PMC8750946

[16] Braam KI Van Dijk-Lokkart EM Van Dongen JM Van Litsenburg RRL Takken T Huisman J Cost-effectiveness of a combined physical exercise and psychosocial training intervention for children with cancer: results from the quality of life in motion study. Eur J Cancer Care. (2017) 26:e12530. 10.1111/ecc.12530 27726229

[17] Wurz A Chamorro-Vina C Guilcher GM Schulte F Culos-Reed SN. The feasibility and benefits of a 12-week yoga intervention for pediatric cancer out-patients. Pediatr Blood Cancer. (2014) 61:1828–34. 10.1002/pbc.25096 24938424

[18] Coombs A Schilperoort H Sargent B. The effect of exercise and motor interventions on physical activity and motor outcomes during and after medical intervention for children and adolescents with acute lymphoblastic leukemia: a systematic review. Crit Rev Oncol Hematol. (2020) 152:103004. 10.1016/j.critrevonc.2020.103004 32580035 PMC8359930

[19] Yeh CH Man Wai JP Lin US Chiang YC. A pilot study to examine the feasibility and effects of a home-based aerobic program on reducing fatigue in children with acute lymphoblastic leukemia. Cancer Nurs. (2011) 34:3–12. 10.1186/s12885-025-15490-1 20706112

[20] Morales JS Santana-Sosa E Santos-Lozano A Baño-Rodrigo A Valenzuela PL Rincón-Castanedo C Inhospital exercise benefits in childhood cancer: a prospective cohort study. Scand J Med Sci Sports. (2020) 30:126–34. 10.1111/sms.13545 31482597

[21] Kuehn M Wypyrsczyk L Stoessel S Neu MA Ploch L Dreismickenbecker E Physical activity as a treatment for cancer-related fatigue in children, adolescents and young adults: a systematic review. Children. (2023) 10(3):572. 10.3390/children10030572 PMC10047895 36980130

[22] Gauss G Beller R Boos J Daggelmann J Stalf H Wiskemann J Adverse events during supervised exercise interventions in pediatric oncology-A nationwide survey. Front Pediatr. (2021) 9:682496. 10.3389/fped.2021.682496 34490156 PMC8417361

[23] Perondi MB Gualano B Artioli GG De Salles Painelli V Filho VO Netto G Effects of a combined aerobic and strength training program in youth patients with acute lymphoblastic leukemia. J Sports Sci Med. (2012) 11:387–92. 24149344 PMC3737942

[24] Blosch C Krombholz A Beller R Gauß G Reinhardt D Götte M. Design and evaluation of an outdoor exercise program for pediatric cancer survivors. Children. (2022) 9:1117. 10.3390/children9081117 35892620 PMC9332767

[25] Kohler BE Sandler CX Baque E Bradford NK Trost SG. Therapeutic exercise interventions in pediatric survivors of brain cancer and other solid tumors: a scoping review. Front Pediatr. (2022) 10:979292. 10.3389/fped.2022.979292 36210932 PMC9535626

[26] Dunn RM Hayes SC Sandler CX Spence RR. Adverse event assessment and reporting in exercise oncology: a review. Exerc Sport Mov. (2023) 1(4):1–7. 10.1249/ESM.0000000000000014

[27] Bernal JDK Recchia F Yu DJ Fong DY Wong SHS Chung PK Physical activity and exercise for cancer-related cognitive impairment among individuals affected by childhood cancer: a systematic review and meta-analysis. Lancet Child Adolesc Health. (2023) 7:47–58. 10.1016/S2352-4642(22)00286-3 36309037

[28] Langer T Grabow D Steinmann D Wörmann B Calaminus G. Late effects and long-term follow-up after cancer in childhood. Oncol Res Treat. (2017) 40:746–50. 10.1159/000484936 29183026

[29] Kroschinsky F Stölzel F Von Bonin S Beutel G Kochanek M Kiehl M Critical Care. (2017) 21:89. 10.1186/s13054-017-1678-1 28407743 PMC5391608

[30] Bo L Wang Y Li Y Wurpel JND Huang Z Chen ZS. The battlefield of chemotherapy in pediatric cancers. Cancers. (2023) 15:1963. 10.3390/cancers15071963 37046624 PMC10093214

[31] Mccarthy E Marchese VG Shipper AG Rock K Felter C. Identifying causes of balance impairment and exploring sensory contributions to balance in pediatric oncology: a scoping review. Crit Rev Oncol Hematol. (2024) 201:104425. 10.1016/j.critrevonc.2024.104425 38909876 PMC11330360

[32] Bölling T Schuck A Pape H Rübe C Meyer FM Martini C Register for the evaluation of side effects after radiation in childhood and adolescence--first results. Klin Padiatr. (2007) 219:139–45. 10.1055/s-2007-973081 17525907

[33] Wilson CL Ness KK. Bone mineral density deficits and fractures in survivors of childhood cancer. Curr Osteoporos Rep. (2013) 11:329–37. 10.1007/s11914-013-0165-0 24043370 PMC4260527

[34] Söntgerath R Eckert K. Impairments of lower extremity muscle strength and balance in childhood cancer patients and survivors: a systematic review. Pediatr Hematol Oncol. (2015) 32:585–612. 10.3109/08880018.2015.1079756 26558954

[35] Gesellschaft für Pädiatrische Onkologie und Hämatologie. S2k-Leitlinie Bewegungsförderung und Bewegungstherapie in der pädiatrischen Onkologie. (2021). Available online at: https://www.awmf.org/uploads/tx_szleitlinien/025-036l_S2k_Bewegungsfoerderung-Bewegungstherapie-in-der-p%C3%A4diatrischen_Onkologie_2021-10.pdf (Accessed February 2, 2026).

[36] Götte M Gauß G Dirksen U Driever PH Basu O Baumann FT Multidisciplinary network ActiveOncoKids guidelines for providing movement and exercise in pediatric oncology: consensus-based recommendations. Pediatr Blood Cancer. (2022) 69:e29953. 10.1002/pbc.29953 36073842

[37] Freites-Martinez A Santana N Arias-Santiago S Viera A. Using the common terminology criteria for adverse events (CTCAE—version 5.0) to evaluate the severity of adverse events of anticancer therapies. Actas Dermosifiliogr. (2021) 112:90–2. 10.1016/j.ad.2019.05.009 32891586

[38] U.S. Department of Health and Human Services. 2017. *Common Terminology Criteria for Adverse Events (CTCAE)*. Available online at: https://ctep.cancer.gov/protocoldevelopment/electronic_applications/docs/CTCAE_v5_Quick_Reference_8.5×11.pdf. 10.3109/15360288.2015.1037530 26095483

[39] Lindgren BM Lundman B Graneheim UH. Abstraction and interpretation during the qualitative content analysis process. Int J Nurs Stud. (2020) 108:103632. 10.1016/j.ijnurstu.2020.103632 32505813

[40] Deckert S Arnold K Becker M Geraedts M Brombach M Breuing J Methodischer standard für die entwicklung von qualitätsindikatoren im rahmen von S3-leitlinien—ergebnisse einer strukturierten konsensfindung. Z Fortbild Qual Gesundhwes. (2021) 160:21–33. 10.1016/j.zefq.2020.11.008 33483285

[41] Thiele C. Abstimmungsverfahren. In: Regeln und Verfahren der Entscheidungsfindung Innerhalb von Staaten und Staatenverbindungen: Staats- und Kommunalrechtliche Sowie Europa- und Völkerrechtliche Untersuchungen. Berlin, Heidelberg: Springer Berlin Heidelberg (2008). 10.1007/978-3-540-78995-6

[42] Smith AR Chein J Steinberg L. Peers increase adolescent risk taking even when the probabilities of negative outcomes are known. Dev Psychol. (2014) 50:1564–8. 10.1037/a0035696 24447118 PMC4305434

[43] Nührenbörger C Mouton C Engelhardt M. Prävention von sportverletzungen im kindes- und jugendalter. Sports Orthop Traumatol. (2021) 37:10–7. 10.1016/j.orthtr.2021.01.005

[44] Nielsen MKF Christensen JF Frandsen TL Thorsteinsson T Andersen LB Christensen KB Effects of a physical activity program from diagnosis on cardiorespiratory fitness in children with cancer: a national non-randomized controlled trial. BMC Med. (2020) 18:175. 10.1186/s12916-020-01634-6 32624004 PMC7336676

[45] Thorsteinsson T Larsen HB Schmiegelow K Thing LF Krustrup P Pedersen MT Cardiorespiratory fitness and physical function in children with cancer from diagnosis throughout treatment. BMJ Open Sport Exerc Med. (2017) 3:e000179. 10.1136/bmjsem-2016-000179 28761697 PMC5530132

[46] Bitsko MJ Cohen D Dillon R Harvey J Krull K Klosky JL. Psychosocial late effects in pediatric cancer survivors: a report from the Children's Oncology group. Pediatr Blood Cancer. (2016) 63:337–43. 10.1002/pbc.25773 26488337 PMC4715481

[47] Schröder HM Lilienthal S Schreiber-Gollwitzer BM Grießmeier B Hesselbarth B Lein-köhler I 2019. S3 Leitlinie Psychosoziale-Versorgung-Paediatrische-Onkologie-Haematologie. Langversion 1.1 ed. Berlin: Arbeitsgemeinschaft der Wissenschaftlichen Medizinischen Fachgesellschaften (AWMF).

[48] Zucchetti G Rossi F Chamorro Vina C Bertorello N Fagioli F. Exercise program for children and adolescents with leukemia and lymphoma during treatment: a comprehensive review. Pediatr Blood Cancer. (2018) 65:e26924. 10.1097/NCC.0b013e3181e4553c 29314654

[49] Neu M Dreismickenbecker E Lanfranconi F Stoessel S Balduzzi A Wright P Get strong to fight childhood cancer—an exercise intervention for children and adolescents undergoing anti-cancer treatment (FORTEe): rationale and design of a randomized controlled exercise trial. BMC Cancer. (2025) 25:1275. 10.1186/s12885-025-14489-y 40775303 PMC12330123

[50] Wurz A Price J Oser M Filiatrault M Conlan H Grimshaw SL. Exploring physical activity experiences among children and adolescents during and beyond cancer treatment: a meta-synthesis of qualitative research. BMC Cancer. (2026) 26:326. 10.1186/s12885-025-15490-1 41629907 PMC12973550

[51] Deutsche Krebsgesellschaft. S3-Leitlinie Komplementärmedizin in der Behandlung von onkologischen PatientInnen. (2024). Available online at: https://register.awmf.org/assets/guidelines/032-055OLl__S3_Komplementaermedizin-in-der-Behandlung-vononkologischen-PatientInnen-2025-06.pdf (Accessed February 2, 2026).

[52] Baumann FT Jensen W Berling-Ernst A Theurich S Leitzmann M Götte M. Exercise therapy in oncology—the impact on quality of life and Side effects. Dtsch Arztebl Int. (2024) 121:331–7. 10.3238/arztebl.m2024.0038 38509786 PMC11413772

[53] Kesting S Weeber P Schönfelder M Renz BW Wackerhage H Von Luettichau I. Exercise as a potential intervention to modulate cancer outcomes in children and adults? Front Oncol. (2020) 10:196. 10.3389/fonc.2020.00196 32154183 PMC7047207

