# Supplementary material for: Incidence, characteristics and suggestions for prevention of adverse events in supervised pediatric oncology exercise sessions
Source: Front Pediatr. 2026 Apr 29;14:1809915. doi: 10.3389/fped.2026.1809915 (PMC13167993; doi:10.3389/fped.2026.1809915)
Supplement: Supplementary Table 1 — Types of triggers contributing to adverse events – definitions and examples. [file Table1.docx]

Table 1: Types of triggers contributing to adverse events: Definitions and examples. (Supplementary Material)

| Trigger | Examples |
| --- | --- |
| Physical (over)exertion | Any form of physical activity that may exceed the patient’s exercise capacity, including running, table tennis, resistance training, stretching, or general full-body exertion. |
| Medical treatment | Symptoms directly associated with medical treatment, such as nausea and vomiting, pain at venous access sites, or mucositis‑related pain. |
| Fall-related incident | Falls resulting from tripping over infusion lines, uneven flooring, objects on the ground, or slipping on wet surfaces; also includes misjudgment of distances or environmental conditions leading to a fall. |
| Coordination problems | Loss of balance or impaired motor control without external influence. |
| Psychological stress | Emotional responses triggered by fear, insecurity, or overwhelming psychological strain in the context of severe illness. |
| Collision | Unintentional contact with other individuals (e.g., during movement games) or with stationary objects such as cabinets or equipment. |
| Environmental conditions | External factors influencing physical (over)exertion, such as mask use due to hygiene protocols, high ambient temperatures, or low oxygen levels in poorly ventilated rooms |
