# Supplementary material for: Incidence, characteristics and suggestions for prevention of adverse events in supervised pediatric oncology exercise sessions
Source: Front Pediatr. 2026 Apr 29;14:1809915. doi: 10.3389/fped.2026.1809915 (PMC13167993; doi:10.3389/fped.2026.1809915)
Supplement: Supplementary Table 2 — Adapted CTCAE criteria for grading the severity of adverse events (AEs). [file Table2.docx]

**Table 2:** Adapted CTCAE criteria for grading the severity of adverse events (AEs). (Supplementary Material)

| CTCAE-Grading | Exercise-specific adapted Common Terminology Criteria for Adverse Events (CTCAE) |
| --- | --- |
| 1 | **Mild**   - Mild; exercise interventions can be continued, no medical treatment is necessary. - The exercise session is interrupted and continued with content or intensity modification; no medical treatment is required. - Symptoms resolve through adjustments to the exercise session (e.g., temporary suspension, reducing intensity, postponing, or changing activity). - Following a strenuous session, participation in other therapeutic activities (e.g., art therapy) may be temporarily not possible due to fatigue (mild limitations in activities of daily living). - Clearance to continue is provided after expert assessment and observation. - Structural consequences rather than patient-related consequences may occur (e.g., adjusting minimum platelet thresholds for participation). - Includes educational or psychological interventions. - Falls are classified as AEs if they result in no or only mild pain; follow-up includes cause clarification (e.g., coordination, weakness). |
| 2 | **Moderate**   - Partial limitation in activities of daily living; ADLs are possible only with pain. - Short recovery period expected; minor medical treatment required. - Participation in ward-based activities (e.g., playroom, art therapy) may be limited due to symptoms such as pain. - Medical rather than pedagogical intervention is required. - Includes medical implementation of the RICE protocol (rest, ice, compression, elevation) when used for treatment of an actual injury. |
| 3 | **Severe**   - Requires invasive or intensive medical treatment. - Increased need for nursing care. - Hospitalization may be necessary. - Significant medical intervention needed. |
| 4 | Life-Threatening Consequence |
| 5 | Death |
