# Supplementary material for: Incidence, characteristics and suggestions for prevention of adverse events in supervised pediatric oncology exercise sessions
Source: Front Pediatr. 2026 Apr 29;14:1809915. doi: 10.3389/fped.2026.1809915 (PMC13167993; doi:10.3389/fped.2026.1809915)
Supplement: Supplementary Table 3 — Categorical summary of the group with completely recorded cases (N = 105) for statistical testing. [file Table3.docx]

**Table 3:** Categorical summary of the group with completely recorded cases (N = 105) for statistical testing. (Supplementary Material)

| **Variable** | **Categories / Definition** | **Notes / Prioritization** |
| --- | --- | --- |
| **Type of AE** | - Injury: superficial, bone, and soft‑tissue injuries - Pain: documented as a standalone category - General symptoms: non‑specific complaints (e.g., circulatory problems, coughing fit, nausea/vomiting, itching, spontaneous/painful bowel movements, physical (over)exertion, psychological stress reactions) | For multiple mentions, pain is documented as a secondary symptom and not as the primary category. Assignment is made to the dominant main category. |
| **Trigger** | - Collision and fall incidents: coordination problems, falls, collisions - Medical treatment: standalone category - Physical (over)exertion: standalone category, includes environmental conditions - Psychological stress reaction: standalone category | Prioritization: medication-related triggers only considered if sole cause; falls take precedence over other categories |
| **Group Size** | - Individual - Group: all group-based training formats regardless of size | Reduced to two categories |
| **Age** | - Children: ≤ 9 years - Adolescents: 10–18 years- Adults: > 18 years | Age groups for analysis and correlation |
| **Motor Performance** | - Coordination: dominant category - Combination: all other multiple mentions + " Multimodal" | If coordination is mentioned, assigned exclusively to this category |
| **CTCAE Grading** | - Grade 1 - Grad 2 and 3 |  |
