# Supplementary material for: Incidence, characteristics and suggestions for prevention of adverse events in supervised pediatric oncology exercise sessions
Source: Front Pediatr. 2026 Apr 29;14:1809915. doi: 10.3389/fped.2026.1809915 (PMC13167993; doi:10.3389/fped.2026.1809915)
Supplement: Supplementary file 7 [file Supplementaryfile1.pdf]

AE-Registry

Please complete the survey below.

Thank you!

[02.01] Date [year_month_day]	__________________________________
[01.01] Assessment_CTCAE Rating	◯ no AE<br>◯ 1<br>◯ 2<br>◯ 3<br>◯ 4<br>◯ 5
[01.02] When did the AE first occur?	◯ First occurence<br>◯ Already present<br>◯ Exercise-related<br>◯ First occurrence and already present
[01.03] Assessment - Do you believe the AE is associated with the sport?	◯ Yes<br>◯ No
[02.02] Basics - What was the AE?	☐ Pain<br>☐ Nausea/Vomiting<br>☐ Circulatory problems<br>☐ Soft tissue/injury<br>☐ Superficial injuries<br>☐ Psychological stress reaction<br>☐ Muscle soreness<br>☐ Severe exhaustion<br>☐ Coughing fit<br>☐ Itching<br>☐ Bone injuries<br>☐ Enuresis<br>☐ Nosebleed<br>☐ Spontaneous painful bowel movement<br>☐ Muscle cramps
Other AE	__________________________________
[02.03] Basics - What caused the AE?	☐ Physical strain<br>☐ Medical therapy<br>☐ Fall event<br>☐ Coordination problems<br>☐ Psychological strain<br>☐ Colliding<br>☐ Environmental conditions<br>☐ Other
Other causes	__________________________________

[02.04] Basics - Which body part was affected?	☐ Internal medicine type<br>☐ Lower extremities<br>☐ Head<br>☐ Abdomen<br>☐ Upper extremities<br>☐ Back<br>☐ Full body<br>☐ Buttocks<br>☐ Chest<br>☐ Intestine<br>☐ Coccyx<br>☐ Intimate area
[06.01] Basics - Detailed description of the occurrence, the trigger, and the nature of the AE.	__________________________________
[03.01] Consequences - Did pain occur?	◯ Yes<br>◯ No<br>◯ Don't know
[03.01.01] Consequences - Pain scale	◯ 1<br>◯ 2<br>◯ 3<br>◯ 4<br>◯ 5<br>◯ 6<br>◯ 7<br>◯ 8<br>◯ 9<br>◯ 10
[03.01.02] Consequences - How long did the pain last?	☐ Pain on the same day<br>☐ Pain until the next day<br>☐ Pain lasting at least 3 days<br>☐ Don't know
[03.02] Consequences - Did hospitalization or an extension of a hospital stay occur?	◯ Yes<br>◯ No<br>◯ Don't know
[03.02.01] Consequences - How soon after the AE was it decided to seek or extend hospitalization?	◯ Within the first 30 Minutes<br>◯ Within the first 24 hours<br>◯ After 24 hours<br>◯ Don't know
[03.02.02] Consequences - How long did the (extended) hospitalization last?	◯ At least 1 day<br>◯ At least 3 days<br>◯ At least 7 days<br>◯ Don't know
[03.03] Consequences - Did medical follow-up care occur?	◯ Yes<br>◯ No<br>◯ Don't know

[03.03.01] Consequences - What did the medical follow-up care involve?	☐ Manual examination<br>☐ Invasive examination<br>☐ Oral medication<br>☐ Intravenous medication<br>☐ Surgery<br>☐ R.I.C.E. protocol<br>☐ Observation<br>☐ Life-saving intervention<br>☐ Desinfection<br>☐ Bandage, plaster<br>☐ Cream and ointment<br>☐ Massage<br>☐ Other<br>☐ Don't know
[07.03] Other: Consequences - What did the medical follow-up care involve?	__________________________________
[03.03.02] Consequences - When did the medical follow-up care take place after the AE?	☐ Within the first 24 hours<br>☐ Within the first week<br>☐ After the first week<br>☐ Don't know
[03.04] Consequences - Was there a significant delay in adhering to the medical treatment protocol?	◯ Yes<br>◯ No<br>◯ Don't know
[03.04.01] Consequences - How was the treatment protocol delayed?	☐ Delay in IV chemotherapy<br>☐ Delay in radiation therapy<br>☐ Delay in surgery<br>☐ Delay in oral chemotherapy<br>☐ Other<br>☐ Don't know
Other: Consequences - How was the treatment protocol delayed?	____________________________
[03.04.02] Consequences - How long was the treatment protocol delayed?	◯ Up to 24 hours<br>◯ Up to 3 days<br>◯ Up to 7 days<br>◯ More than 7 days<br>◯ Don't know
[03.05] Consequences - Were life-saving interventions (e.g., ventilation, CPR) necessary?	◯ Yes<br>◯ No<br>◯ Don't know
[03.05.01] Consequences - What life-saving interventions were carried out?	☐ Cardiopulmonary resuscitation<br>☐ Defibrillation<br>☐ Emergency medications (e.g., adrenaline, nitroglycerin)<br>☐ Blood transfusion(s)<br>☐ Emergency surgery (e.g., trauma surgery)<br>☐ Ventilation/oxygen therapy<br>☐ Other<br>☐ Don't know
Other: Consequences - What life-saving interventions were carried out?	____________________________

[03.05.01] Consequences - How often was the life-saving intervention repeated?	◯ Once<br>◯ Up to 5 times<br>◯ More than 10 times<br>◯ Don't know
[03.06] Consequences - Was there an increased need for care?	◯ Yes<br>◯ No<br>◯ Don't know
[03.06.01] Consequences - In which areas was the increased care required?	☐ Mobility (e.g., assistance with dressing and movement, wheelchair use)<br>☐ Personal hygiene (e.g., help with washing, brushing teeth, dressing)<br>☐ Medical care (e.g., therapies and medication)<br>☐ Nutrition (e.g., tube feeding, special diets)<br>☐ Psychosocial support (e.g., emotional support, behavioral interventions)<br>☐ Other
Other: Consequences - In which areas was the increased care required?	__________________________________
[03.06.02] Consequences - How long did the increased need for care last?	◯ Up to 3 hours<br>◯ Up to 24 hours<br>◯ Up to 48 hours<br>◯ Over 2 days<br>◯ Over 1 week<br>◯ Don't know
[03.07] Consequences - Were medications administered?	◯ Yes<br>◯ No<br>◯ Don't know
[03.07.01] Consequences - What medications were administered?	☐ Analgesics (painkillers)<br>☐ Antibiotics (for bacterial infections)<br>☐ Antiviral medications (for viral infections)<br>☐ Antidepressants (for depression)<br>☐ Antipsychotics (neuroleptics)<br>☐ Antiepileptic drugs (for epilepsy)<br>☐ Antiallergic drugs (for allergies)<br>☐ Anticoagulants (blood thinners)<br>☐ Other
[07.04]: Other: Consequences - What medications were administered?	__________________________________
[03.07.02] Consequences - How long were these medications administered?	◯ Only once<br>◯ Up to 3 days<br>◯ Up to 7 days<br>◯ More than 7 days<br>◯ Don't know
[03.08] Consequences - Did anxiety or uncertainty arise?	◯ Yes<br>◯ No<br>◯ Don't know

[03.08.01] Consequences - Who experienced anxiety or uncertainty?	☐ The affected individual<br>☐ The parents of the affected individual<br>☐ The treatment team<br>☐ The exercise specialist<br>☐ Refusal of further therapeutic sports activities
[03.08.02] Consequences - How long did anxiety or uncertainty influence the situation?	◯ Only on the same day<br>◯ Until the end of the week<br>◯ Until the end of the month<br>◯ Forever
[03.09] Consequences - Were structural adjustments made?	◯ Yes<br>◯ No<br>◯ Don't know
[03.09.01] Consequences - What structural adjustments were made?	◯ Spatial adjustments<br>◯ Personnel adjustments<br>◯ Protective equipment<br>◯ Rule changes<br>◯ Training structure<br>◯ Increased mindfulness<br>◯ Other
Other structural adjustments	__________________________________
[03.09.02] Consequences - How long did the structural adjustments last?	◯ Only on the same day<br>◯ Until the end of the week<br>◯ Until the end of the month<br>◯ No longer
[03.10] Consequences - Was the situation reviewed by an expert?	◯ Yes<br>◯ No
[03.10.01] Consequences - Who reviewed the situation?	◯ Parents<br>◯ Nursing staff<br>◯ Medical professionals<br>◯ Psychosocial services<br>◯ Physical therapy<br>◯ Other
[07.05]: Other: Consequences - Who reviewed the situation?	__________________________________
[03.10.02] Consequences - Was approval given to proceed?	◯ Yes<br>◯ No<br>◯ Don't know
[03.11] Consequences - Was the R.I.C.E. protocol applied?	◯ Yes<br>◯ No<br>◯ Don't know
[03.11.01] Consequences - Which specific components of the R.I.C.E. protocol were applied?	☐ Rest<br>☐ Ice<br>☐ Compression<br>☐ Elevation

[03.11.02] Consequences - How long was the R.I.C.E. protocol applied?	◯ Up to 3 hours<br>◯ Up to 24 hours<br>◯ Up to 7 days<br>◯ More than 7 days<br>◯ Don't know
[03.12] Consequences - Was the child observed after the AE?	◯ Yes<br>◯ No<br>◯ Don't know
[03.12.01] Consequences - How was the observation carried out?	☐ Continuous monitoring (e.g., intensive care unit)<br>☐ Vital sign monitoring (e.g., regular measurement of heart rate, blood pressure, respiratory rate, body temperature, and oxygen saturation)<br>☐ Documentation (careful recording of health condition)<br>☐ Observation of specific symptoms (e.g., neurological observations)<br>☐ Technological support (e.g., monitoring systems such as EKG)<br>☐ Other
[07.07] Other: Consequences - How was the observation carried out?	__________________________________
[03.12.02] Consequences - How long was the child observed?	◯ Up to 3 hours<br>◯ Up to 24 hours<br>◯ Up to 7 days<br>◯ More than 7 days<br>◯ Don't know
[03.13] Consequences - Was the exercise session stopped?	◯ Yes<br>◯ No
[03.13.01] Consequences - How was the session stopped?	◯ Pause<br>◯ Termination
[03.13.02] Consequences - How long was the pause?	◯ Up to 1 minute<br>◯ Up to 5 minutes<br>◯ More than 10 minutes
[03.14] Consequences - Was the content of the exercise session changed or adapted?	◯ Yes<br>◯ No
[03.14.01] Consequences - How was the exercise session adapted?	☐ Exercise selection<br>☐ Intensity<br>☐ Equipment<br>☐ Space<br>☐ Motivation strategy<br>☐ Involvement of family members<br>☐ Use of BORG scale<br>☐ Communication strategy<br>☐ Other
Others: Consequences - How was the exercise session adapted?	______________________________

[03.14.02] Folgen - Wie lange wurde die Anpassung vorgenommen?	☐ Only for this session<br>☐ For the entire therapy phase<br>☐ From now on, for all exercise sessions with all patients<br>☐ For the next sessions (AE already happended)
[03.15] Consequences - Was active comforting provided?	○ Yes<br>○ No
[03.15.01] Consequences - Was the approach to handling the AE purely pedagogical?	☐ Yes<br>☐ No
[03.15.02] Consequences - How long was the comforting provided?	○ Up to 1 minute<br>○ Up to 10 minutes<br>○ More than 10 minutes
[03.16] Consequences - Did the AE result in death?	○ Yes<br>○ No
[03.17] Consequences - Were there any restrictions in activities of daily living (ADL)?	○ Yes<br>○ No<br>○ Don't know
[03.17.01] What limitations in activities of daily living (ADL) occurred?	☐ Participation in programs such as art therapy, etc. was not possible.<br>☐ Length of stay increased.<br>☐ Other
Other	______________________________
[03.17.02] Consequences - Duration?	○ At least 24 hours<br>○ At least 3 days<br>○ At least one week
[03.18] [03.17] Consequences - Are there any other relevant consequences?	______________________________________
[05.01] Context - In which phase of therapy did the AE occur?	○ Acute therapy<br>○ Long-term therapy<br>○ Aftercare<br>○ Palliative<br>○ Don't know
[05.02] Context - What was the group size during the exercise session in which the AE occurred?	○ Individual<br>○ Group 2-5<br>○ Group 5 to 10<br>○ Group over 10
[05.03] Context - How old was the affected individual at the time of the AE?	○ < 5 years<br>○ 6 to 9 years<br>○ 10 to 14 years<br>○ 15 to 18 years<br>○ >18 years
[05.04] Context - Did the exercise program take place online?	○ Yes<br>○ No

[05.05] Context - Did the AE occur during a sports test?	◯ Yes<br>◯ No
[05.06] Context - In which setting did the AE occur?	◯ Gym<br>◯ Patients room<br>◯ Hospital corridor<br>◯ Outside<br>◯ At home (via telemedicine)<br>◯ Swimmping pool<br>◯ Others
Other: Context - In which setting did the AE occur?	__________________________________
[05.07] Context - Which primary motor form dominated the exercise session?	☐ Coordination<br>☐ Endurance<br>☐ Strength<br>☐ Flexibility<br>☐ Full body<br>☐ Speed<br>☐ Relaxation<br>☐ Other
Other: Context - Which primary motor form dominated the exercise session?	__________________________________
[05.08] Context - During which phase of the planned exercise session did the AE occur?	◯ 1st half<br>◯ 2nd half<br>◯ Don't know
[05.09] Context - What was the fitness level of the affected individual when the AE occurred?	☐ Good<br>☐ Average<br>☐ Moderate<br>☐ Don´t know<br>☐ Note
[06.11] Context - Note	__________________________________
[06.12] Basics - Other?	____
